# σ^E^ controlled regulation of porin OmpU in *Vibrio cholerae*

**DOI:** 10.1111/mmi.14669

**Published:** 2021-01-25

**Authors:** Nina Pennetzdorfer, Thomas Höfler, Martina Wölflingseder, Sarah Tutz, Stefan Schild, Joachim Reidl

**Affiliations:** ^1^ Institute of Molecular Biosciences University of Graz Graz Austria; ^2^ BioTechMed‐Graz Graz Austria; ^3^ Field of Excellence BioHealth University of Graz Graz Austria

**Keywords:** OmpU, RpoE, stress response, ToxR, transcriptional regulation and *Vibrio cholerae*

## Abstract

Bile resistance is essential for enteric pathogens, as exemplified by *Vibrio cholerae*, the causative agent of cholera. The outer membrane porin OmpU confers bacterial survival and colonization advantages in the presence of host‐derived antimicrobial peptides as well as bile. Expression of *ompU* is controlled by the virulence regulator ToxR. *rpoE* knockouts are accompanied by suppressor mutations causing *ompU* downregulation. Therefore, OmpU constitutes an intersection of the ToxR regulon and the σ^E^‐pathway in *V*. *cholerae*. To understand the mechanism by which the sigma factor σ^E^ regulates OmpU synthesis, we performed transcription studies using *ompU* reporter fusions and immunoblot analysis. Our data revealed an increase in *ompU* promoter activity in Δ*rpoE* strains, as well as in a Δ*ompU* background, indicating a negative feedback regulation circuit of *ompU* expression. This regulation seems necessary, since elevated lethality rates of Δ*rpoE* strains occur upon *ompU* overexpression. Manipulation of OmpU’s C‐terminal portion revealed its relevance for protein stability and potency of σ^E^ release. Furthermore, Δ*rpoE* strains are still capable of elevating OmpU levels under membrane stress conditions triggered by the bile salt sodium deoxycholate. This study provides new details about the impact of σ^E^ on *ompU* regulation, which is critical to the pathogen’s intestinal survival.

## Graphical abstract

A regulatory circuit indicates the interplay between the major outer membrane porin OmpU, periplasmic stress stimuli and the σE response pathway in Vibrio cholerae. This study reveals a negative feedback regulation resulting in fine‐tuning of the ompU expression level, balancing stress response and survival. Putative factor(s) operating on the negative feedback regulation on ompU expression is shown in burgundy colour.

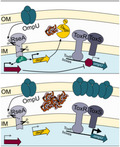

## 1 INTRODUCTION

*Vibrio cholerae*, a Gram‐negative, facultative anaerobic bacterium, is an infectious agent that causes the human disease cholera (Cava, 2017). This pathogen is endemic in India, Bangladesh, Southeast Asia, and South America (Faruque et al., 1998). Recent outbreaks in Haiti and Yemen have shown its high spreading potential (Ramamurthy et al., 2019). The estimated number of cholera cases worldwide reaches 2.9 million, with 95.000 deaths every year (Legros, 2018). The bacterium is able to transit between two environmental habitats, an aquatic reservoir, where it persists by forming biofilms on chitinous surfaces of crustaceans, zooplankton, and phytoplankton, and the human gastrointestinal tract (Colwell, 2004). Upon oral ingestion of contaminated water or food, *V*. *cholerae* colonizes the small intestine after passage of the gastric acid barrier and the penetration of the intestinal epithelial mucus lining. During the passage through the stomach to its primary site of infection, the pathogen rapidly adapts its physiology by altering gene expression, protein biosynthesis and stability, posttranscriptional control, and surface exchange in order to counteract host‐specific stress responses. Eventually, virulence gene expression is induced, enforcing full colonization fitness (Cakar et al., 2018; DiRita et al., 1991; Herrington et al., 1988; Pennetzdorfer et al., 2019; Zingl et al., 2020). Stressors attacking the bacterial outer membrane (OM) interfere with *V*. *cholerae* survival in the intestine. Bile salts, organic acids, factors of the innate immune system, for example complement factors secreted by intestinal epithelial cells (Andoh et al., 1998) and defensins produced by Paneth cells (Mallow et al., 1996), are ranked among such membrane‐disruptive agents.

The pathogenesis of cholera is primarily based on the activity of secreted cholera toxin (CT) and the production of toxin‐coregulated pili (TCP), eventually resulting in a highly severe form of secretory diarrhea (Childers and Klose, 2007). This virulence gene cascade termed the ToxR regulon, consists of several transcription factors, for example the transcriptional complex ToxRS, which is integrated into the inner membrane (Peterson and Gellings, 2018). In addition to the initiation of the ToxR regulon, ToxRS inversely regulate the expression of virulence‐associated outer membrane porins (OMPs) encoded by *ompU* and *ompT*. Upon host entry, the activation of *ompU* transcription and *ompT* repression by ToxR, as well as the exchange of OmpT for OmpU, are crucial for the rapid establishment of bile salt resistance, facilitating *V*. *cholerae* to colonize and survive within the human intestine (Crawford et al., 1998; Li et al., 2000; Provenzano and Klose, 2000; Zingl et al., 2020). Promoter regions of both genes harbor AT‐rich ToxR binding elements. This 5′‐TNAAA‐N_5_‐TNAAA‐3′ element (or its reverse complement) occurs once upstream of the *ompT* coding sequence (CDS) and three times at the *ompU* operator site (Goss et al., 2013). Concordantly, the bile salt sodium deoxycholate (Na‐DC) modulates OMP regulation by activating the ToxRS complex in both O1 biotypes El Tor and classical strains (Lembke et al., 2018; Provenzano and Klose, 2000; Provenzano et al., 2000). Additionally, increased ToxR levels achieved in response to supplementation with the amino acids asparagine, arginine, glutamate, and serine (NRES) were shown to positively affect *ompU* activation and *ompT* repression (Mey et al., 2012), whereas higher temperatures cause an opposite regulatory effect (Parsot and Mekalanos, 1990).

Electrophysical channel characterization revealed various differences between OmpT and OmpU. In contrast to OmpT, OmpU possesses higher cation selectivity, leading to an influx restriction of negatively charged components across the OM, and consequently preventing cytoplasmic membrane destruction (Pathania et al., 2018; Simonet et al., 2003). Although bile resistance acquired by OmpU might be indirect, *ompU* or *toxR* deletion strains are more susceptible to bile salt damage than strains lacking OmpT in their OMs. Since bile salt uptake across the OM by OmpU is lower than that by OmpT, *ompT* repression is more important than *ompU* expression (Wibbenmeyer et al. 2001).

Moreover, initiation of regulated intramembrane proteolysis (RIP) of ToxR under stress conditions is inhibited in the presence of Na‐DC, ensuring ToxR‐operator binding by enhanced homo‐ and heterodimer formation between ToxR and ToxS (Lembke et al., 2018; Lembke et al., 2020; Midgett et al., 2017).

Additionally, OmpU was identified to act as a signal transducer in the σ^E^ pathway (Mathur et al., 2007). In general, this system facilitates the response of Gram‐negative bacteria to misfolded periplasmic proteins and perturbation of the OM (Mescas et al., 1993; Ruiz and Silhavy, 2005). The alternative sigma factor σ^E^ (encoded by *rpoE*) is sequestered to the inner side of the cytoplasmic membrane by the membrane‐embedded anti‐sigma factor RseA to keep it inactive in unstressed cells (De Las Penas et al., 1997; Missiakas et al., 1997). Exposure of C‐terminal signal peptides of misfolded OMP to the periplasm results in PDZ domain‐dependent activation of the site‐1 protease DegS and, in turn, site‐2 protease RseP/YaeL, initiating RIP of RseA (Alba et al., 2002; Griorova et al., 2004; Kanehara et al., 2002; Walsh et al., 2003; Wilken et al., 2004). Thus, σ^E^ associated with residual RseA protein is released into the cytoplasm, where RseA remnants are degraded by ClpXP (Flynn et al., 2004). Eventually, σ^E^ can interact with RNA polymerase core enzyme to initiate transcription from σ^E^‐dependent promoters (De Las Penas et al., 1997; Missiakas et al., 1997). Genes belonging to the σ^E^ regulon encode for *rpoE* itself and for periplasmic chaperones and proteases, for example *degP*, which supports refolding or acts as a protease (Dartigalongue et al., 2001; Kabir et al., 2005; Kovacikova and Skorupski, 2002; Krojer et al., 2002; Rhodius et al., 2006; Spiess et al., 1999).

In *V*. *cholerae*, the σ^E^ RIP system is linked to the ToxR regulon, as exemplified by proteolysis of the membrane‐bound transcription factors TcpP and ToxR affecting virulence gene expression (Pennetzdorfer et al., 2019). Based on current models, OmpU activated by ToxR acts as a sensor component, and comprises a C‐terminal signal motif that can be recognized by the DegS PDZ domain upon exposure to human defensin P2 or polymyxin B (Mathur et al., 2007). High‐throughput sequence analysis of *rpoE* deletion strains revealed suppressor mutations within the *ompU* transcriptional control region, resulting in reduced *ompU* expression (Davis and Waldor, 2009).

Intriguingly, OmpU seems to constitute an intersection point of the ToxR regulon and σ^E^ pathway, as evidenced by suppressor mutations originated within the *ompU* promoter upstream region. By using transcriptional reporter fusions, we demonstrate that σ^E^ release depends on OmpU, but proper *ompU* expression also requires σ^E^ and OmpU itself. Remarkably, overexpression of *ompU* is detrimental to Δ*rpoE* strains, resulting in significant survival deficiency. Additionally, our data highlight the importance of the OmpU C‐terminal peptide in σ^E^ activation and OmpU stability. In this context, we provide evidence for the molecular mechanisms of a negative feedback loop in order to maintain OmpU at physiologically operating levels in *V*. *cholerae*.

## 2 RESULTS

### 2.1 *ΔompU* strains tolerate *rpoE* deletions

**FIGURE 1:**
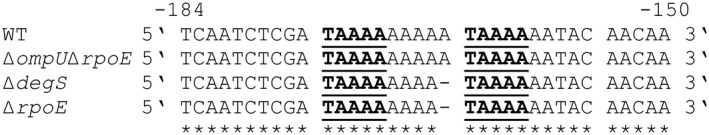
Position of suppressor mutations within the ToxR binding box. Multiple DNA‐sequence alignment in ClustalW format (5′–3′) of ToxR binding box (O1) −184 to −150 bp upstream to the transcription start site +1 of *ompU* in WT, Δ*ompU*Δ*rpoE*, Δ*degS* and Δ*rpoE*. Single nucleotide deletions in Δ*degS* and Δ*rpoE* strains are marked by a hyphen; identical nucleotides are highlighted by asterisks. ToxR binding boxes are displayed in bold and underlined as described in Crawford et al. (1998)

The alternative sigma factor σ^E^ is well known in *Escherichia coli*, and it is strictly linked to OM stress induced by high temperatures or OMP overproduction (Erickson and Gross, 1989; Mecsas et al., 1993). Previous work reported that the homologue of *rpoE* in *V*. *cholerae* is not essential for survival (Ding et al., 2004; Kovacikova and Skorupski, 2002). Later findings revealed suppressor mutations in the upstream region of the *ompU* CDS in a *rpoE* deletion background (Davis and Waldor, 2009). Therefore, we hypothesized that Δ*ompU* strains tolerate *rpoE* knockouts, and sequenced the entire operator and promoter regions of *ompU*, including the 5′ untranslated region (UTR) of WT, Δ*ompU*Δ*rpoE*, several Δ*rpoE*, and Δ*degS* strains generated under the same selective growth conditions (Figure 1). The isolated strains, either lacking the entire *rpoE* or *degS* gene, each revealed a single nucleotide deletion within the spacer region of the two ToxR boxes in operator O1 compared to the WT. In contrast, deletion of *rpoE* in the Δ*ompU* strain resulted in an unaffected *ompU* operator sequence. It is worth noting that DegS is also required for counteracting membrane stress in *V*. *cholerae* when challenged with cationic antimicrobial peptides (Mathur et al., 2007). Since *degS* mutants cannot liberate σ^E^ from the membrane, they cause similar consequences as *rpoE* knockout strains. Taken together, *rpoE* and *degS* deletion strains show suppressor mutations in the *ompU* expression control region, whereas no suppressor mutations were observed in Δ*ompU* strains.

### 2.2 *ΔrpoE* strains can still react to bile salts by elevating OmpU levels

**FIGURE 2:**
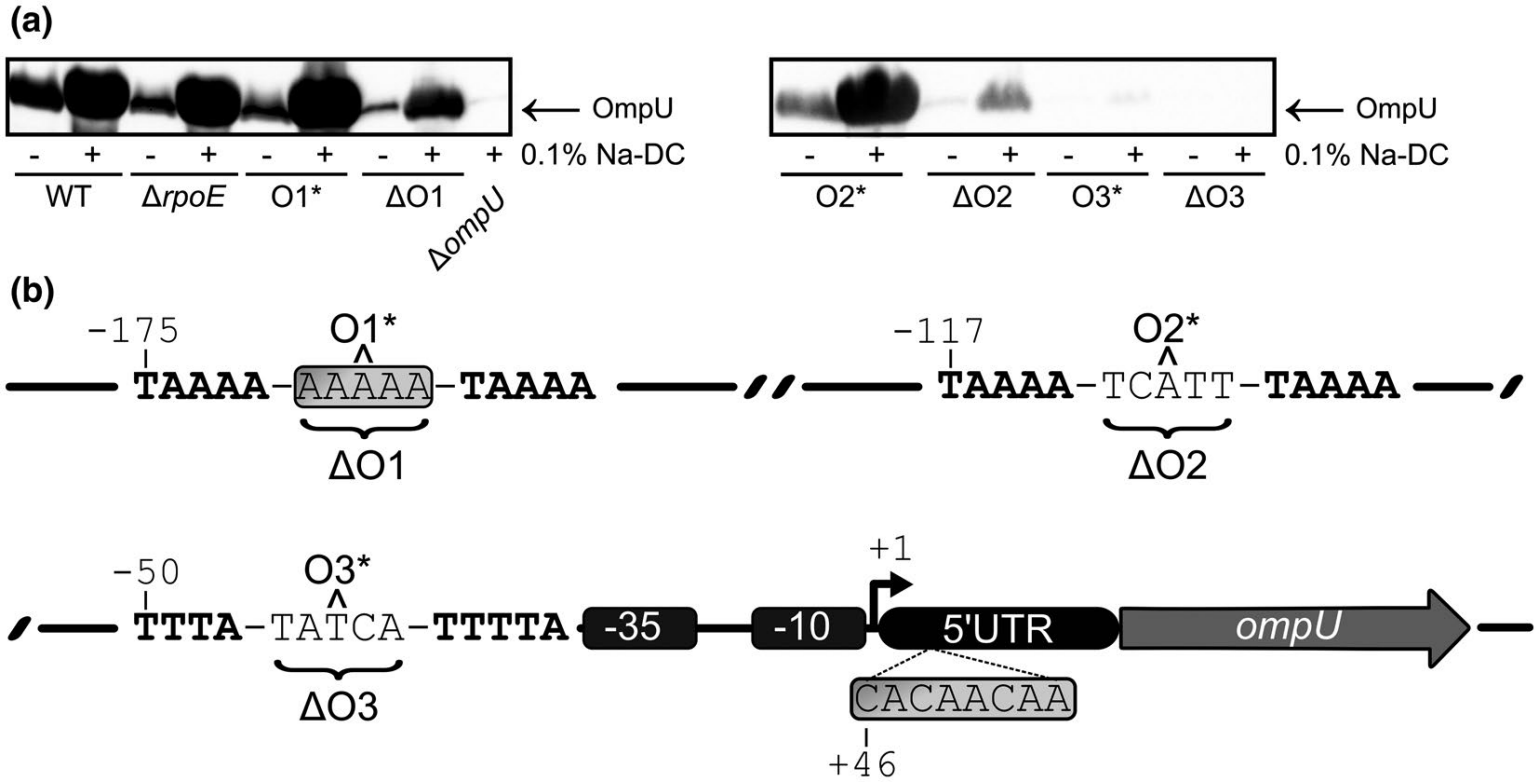
Analysis of bile salt‐dependent changes in OmpU‐ and ToxR levels in *ompU* operator mutants. (a) Immunoblot analysis of WCLs obtained from WT, Δ*rpoE*, *ompU*O1*, *ompU*ΔO1, *ompU*O2*, *ompU*ΔO2, *ompU*O3*, *ompU*ΔO3 and Δ*ompU* utilizing α‐OmpU antibodies grown in LB supplemented without or with 0.1% Na‐DC ON. (b). Operator regions with respective ToxR binding boxes are displayed in bold each with the respective spacing sequences of 5 bp. Promoter elements are drawn to scale to the transcription start site (+1) and are highlighted in dark grey. The 5′UTR is shown as an elliptical black shape, followed by the *ompU* CDS displayed as a light grey arrow, corresponding to the sequence published in (Sperandio et al., 1996). Light grey boxed sequences represent the loci of suppressor mutations: operator O1 was affected either by an insertion or a deletion of a single adenine within its spacing region found in four independently generated Δ*rpoE* strains. Furthermore, we characterized an insertion of 5′CAC AAC AA3′ at position +46 of *ompU* 5′UTR that corresponds to a duplication. To understand the relevance of each single operator, artificial point mutations and deletions of respective operator regions were inserted into the chromosome which were marked by an asterisk or by curved parenthesis, respectively

Since the upstream region of the *ompU* promoter is a target for suppressor mutations, we further characterized the *ompU* promoter control region, focusing on ToxR boxes/operators (Figure 2a). Therefore, and in accordance with previous reports (Davis and Waldor, 2009), we additionally characterized mutations within the *ompU* operator region in independently generated Δ*rpoE* strains, all of which featured either insertions or deletions of single base pairs within ToxR box O1 or O2 (Figure 2b). *ompU* transcription activation directly relies on the membrane located regulator ToxR (Crawford et al., 1998). Three operator sites are located upstream of the transcription start site of *ompU* for ToxR‐specific binding (Crawford et al., 1998). In the presence of Na‐DC, ToxR activates *ompU* expression, resulting in bile resistance of *V*. *cholerae* (Lembke et al., 2018; Provenzano and Klose, 2000).

We therefore asked next whether supplementation with Na‐DC affects OmpU levels in Δ*rpoE* or *ompU*O1* strains carrying a single nucleotide deletion that resulted in space interference between the two ToxR boxes. Equal amounts of protein of whole cell lysates (WCLs) obtained from WT, Δ*rpoE*, and *ompU*O1* cultures incubated in LB supplemented with or without Na‐DC for immunoblot analysis were separated by polyacrylamide gel electrophoresis (Figure S1a,b), and analyzed using α‐OmpU (Figure 2a) or α‐ToxR (Figure S1c) antibodies (Fan et al., 2014; Salem et al., 2015). ToxR levels were similar in all mutant strains cultivated in media containing Na‐DC. On the contrary, strains harboring *ompU*O1* mutations still displayed lower OmpU amounts in LB compared to the WT. In contrast, Δ*rpoE* and *ompU*O1* produced OmpU equally well as the WT, when incubated in LB supplemented with Na‐DC. Additionally, the Δ*rpoE* strain revealed a similar survival decrease upon incubation with 0.1% Na‐DC as the WT (Figure S1d).

We further asked whether the operators had distinct roles in bile‐dependent *ompU* transcription initiation. To address this question, we constructed artificial deletions, either by complete removal of the spacer region between ToxR boxes within one operator site, or by creating spacing interference between two ToxR boxes by single nucleotide deletion. Subsequently, we monitored OmpU (Figure 2a) and ToxR levels (Figure S1c) by immunoblot analysis of WCLs of these strains after incubation in LB with or without Na‐DC, and also performed loading controls (Figure S1a,b). ToxR levels remained at comparable levels regardless of Na‐DC addition. Moreover, *ompU*ΔO1 and *ompU*O2* were still able to mediate a response to Na‐DC with proportionally higher OmpU levels compared to the respective untreated samples. Furthermore, these mutants also displayed reduced OmpU levels compared to WT‐derived OmpU levels produced upon bile salt exposure. In addition, *ompU*ΔO2 revealed a massive attenuation in bile‐dependent OmpU production. Interestingly, both mutations in operator O3 resulted in an entire loss of OmpU at any condition tested. Thus, strains with an *ompU*O1* genotype are still capable of elevating OmpU levels upon bile exposure. In contrast, other mutated operator variants are limited in OmpU production, highlighting operator O3, which is unable to initiate *ompU* transcription, even though ToxR is present.

### 2.3 σ^E^‐mediated transcriptional control of *degP* depends on OmpU

DegP is a multimeric periplasmic protein, which can act as either a protease or chaperone (Spiess et al., 1999). Each DegP subunit provides a PDZ domain, similar to the membrane‐embedded site‐1 protease DegS, inducing its protease activity upon interaction with the protease active center (Kolmar et al., 1996; Krojer et al., 2008).

**FIGURE 3:**
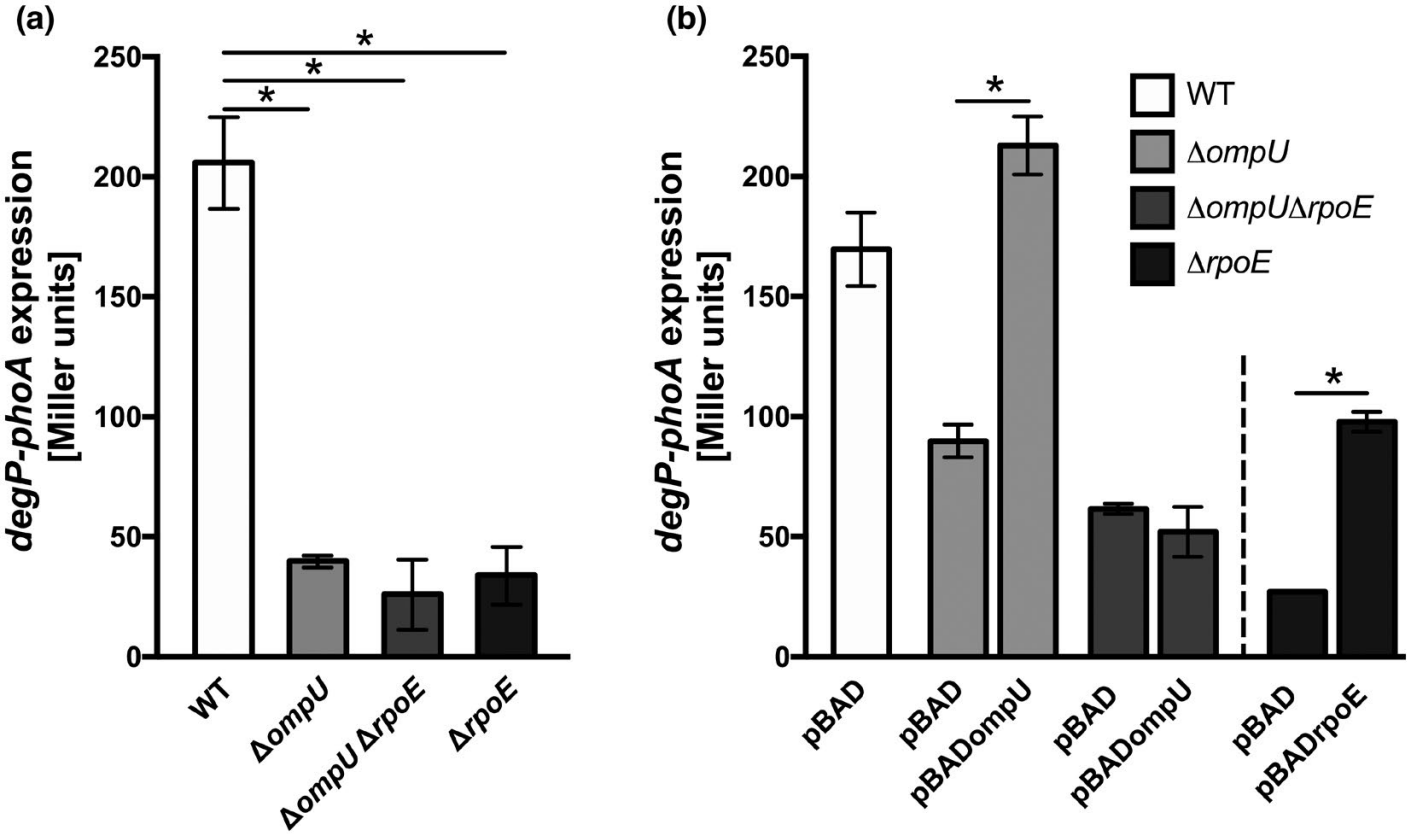
OmpU dependency of *degP* expression. (a) Alkaline phosphatase activities (Miller units) were quantified in WT, Δ*ompU*, Δ*ompU*Δ*rpoE* and Δ*rpoE* harboring a *degP‐phoA* transcriptional fusion grown in LB ON. Data represent mean values ± standard deviation of six biological replicates and a total sample size of 24. Significant differences between the WT and respective deletion strains are indicated by an asterisk (one‐way ANOVA followed by a Dunnett’s multiple comparisons test, *p < .05). (b) Alkaline phosphatase activities (Miller units) of *degP‐phoA* transcriptional fusion were measured in strain backgrounds of WT pBAD, Δ*ompU* pBAD, Δ*ompU* pBADompU, Δ*ompU*Δ*rpoE* pBAD, Δ*ompU*Δ*rpoE*pBADompU, Δ*rpoE* pBAD and Δ*rpoE* pBADrpoE (synonym for pBAD18‐Kanbla^‐^rpoE‐FLAGrseABC), see Table 1. After 2 h of induction with 0.05% arabinose in LB in mid‐log phase, cell samples were harvested and measured. Data represent mean values ± standard deviation of six biological replicates and a total sample size of 12. Significant differences are indicated by an asterisk (one‐way ANOVA followed by a Sidak’s multiple comparisons test, *p < .05)

A microarray study indicated that *degP* expression is dependent on σ^E^ release in *V*. *cholerae* (Ding et al., 2004). Consequently, we used *degP* transcription as a readout for σ^E^ activation to further characterize the link between OmpU and the σ^E^ pathway. To validate differences in *degP* regulation in an OmpU‐dependent manner, we measured alkaline phosphatase (PhoA) activities in *degP‐phoA* reporter strains harboring a transcriptional fusion of the promoterless *phoA* gene to intact *degP* (Figure 3a). Upon deletion of *ompU*, a significant decrease in PhoA activity was observed, which was similar to that observed in the Δ*rpoE* and Δ*ompU*Δ*rpoE* strains.

To further elucidate the role of OmpU in σ^E^ pathway activation, we performed complementation studies of either *ompU* or *rpoE* using Δ*ompU*, Δ*ompU*Δ*rpoE*, and Δ*rpoE* strains, respectively. In these strains, we monitored *degP* expression upon plasmid‐derived induction of *ompU* expression for 2 h in LB medium in mid‐log phase compared to the vector control cultivated under the same conditions (Figure 3b).

A significant elevation in PhoA activity occurred upon *ompU* expression in *trans* compared to the empty vector control with Δ*ompU* background. On the contrary, PhoA activities remained at low level in Δ*ompU*Δ*rpoE* cells upon expression of *ompU* in *trans*, indicating that *degP* expression in such strain background shows co‐dependency on RpoE and OmpU. Moreover, *rpoE* overexpression in the Δ*rpoE* strain led to significantly elevated PhoA activities compared to the respective empty vector control, indicating complementation. Furthermore, significantly reduced PhoA activities were also observed in Δ*degS* grown in LB (Figure S2). Thus, *degP* expression and, therefore, the activation of the σ^E^ pathway directly correlate with the absence or presence of OmpU.

### 2.4 Lethality caused by *ompU* overexpression in *ΔrpoE* strains can be rescued by DegP

**FIGURE 4:**
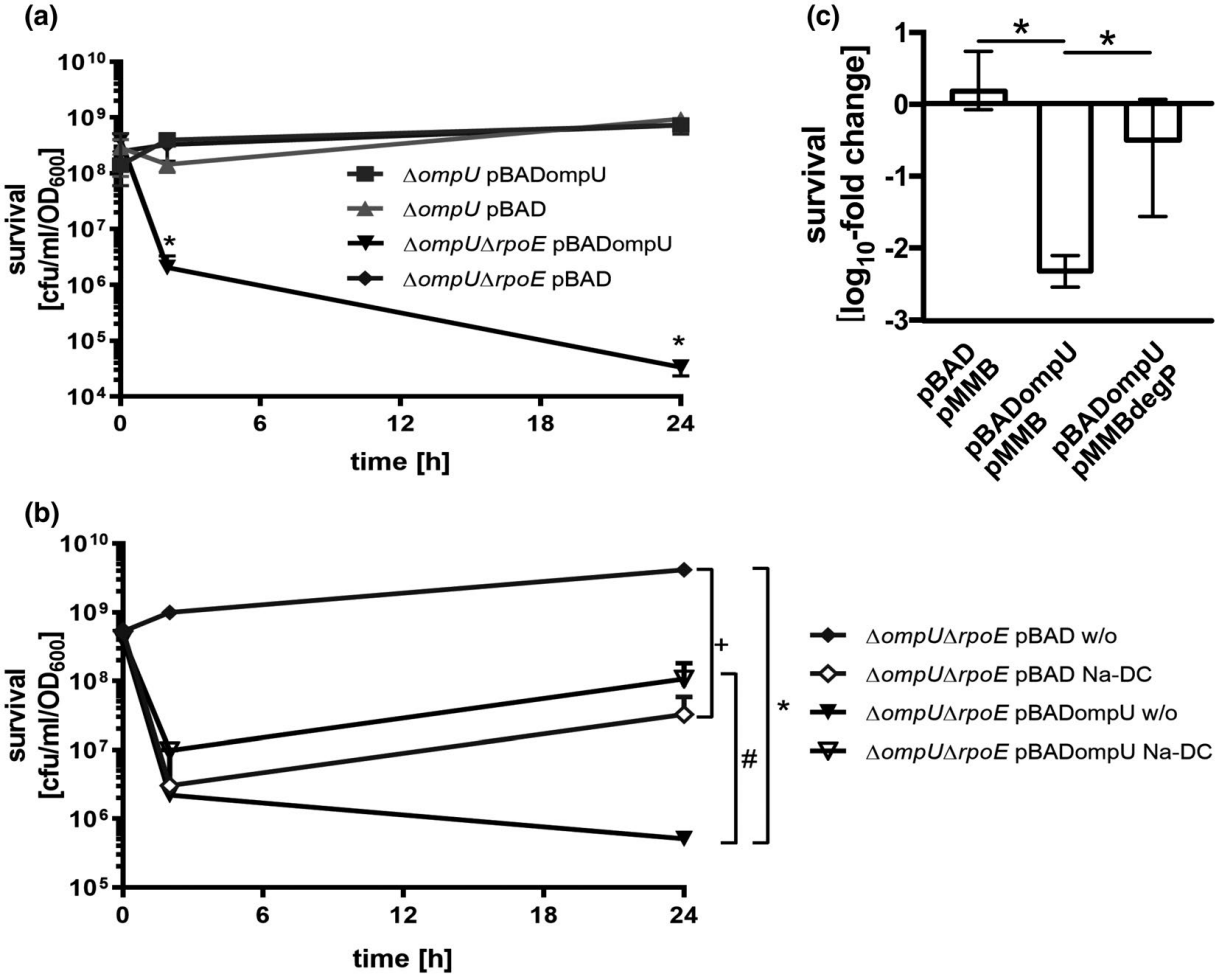
OmpU toxicity in Δ*rpoE* and rescue by DegP. (a). Survival plating of Δ*ompU* and Δ*ompU*Δ*rpoE* carrying pBAD or pBADompU, respectively, was performed after induction of plasmid‐derived expression with 0.05% arabinose in mid‐log phase at time points 0, 2, and 24 h. (B). Shown are survival plating of strains Δ*ompU*Δ*rpoE* carrying pBAD or pBADompU under similar conditions as used in A) except that Na‐DC (0.1%) was added. Data in Figure A and B represent median values ± interquartile range of four biological replicates and a total sample size of at least 10. Significant differences (*p* < .05) between data sets at a given time point are marked by a symbol for the following comparisons: in panel A * Δ*ompU*Δ*rpoE* pBAD w/o pBAD versus Δ*ompU*Δ*rpoE* pBADompU; in panel B * Δ*ompU*Δ*rpoE* pBAD w/o versus Δ*ompU*Δ*rpoE* pBADompU w/o, + Δ*ompU*Δ*rpoE* pBAD w/o versus Δ*ompU*Δ*rpoE* pBAD Na‐DC, # Δ*ompU*Δ*rpoE* pBADompU w/o versus Δ*ompU*Δ*rpoE* pBADompU Na‐DC (Kruskal–Wallis test followed by Dunn’s multiple comparisons test; for a representative loading and quality control, see Kang‐stained gel in Figures S3A). C. log_10_ fold change presentation of survival plating of time point 2 h compared to time point 0 h obtained from Δ*ompU*Δ*rpoE* harboring pBAD and pMMB, pBADompU and pMMB, or pBADompU and pMMBdegP, respectively, was conducted after induction of plasmid‐derived expression with 0.05% arabinose and 1 mM IPTG in mid‐log phase. Data represent median with interquartile range of four biological replicates and a total sample size of 4. Significant differences are indicated by an asterisk (Kurskal–Wallis multiple comparison, *p < .05; for a representative loading and quality control, see Kang‐stained polyacrylamide gel in Figures S4A)

Viable Δ*rpoE* strains are only obtainable with accompanying suppressor mutations in the *ompU* expression control regions. Such mutations lead to artificial downregulation of *ompU* expression phenotypes. To characterize such strains for an *ompU* dependent growth deficiency, we compared the survival fitness of Δ*ompU* to Δ*ompU*Δ*rpoE* upon induction of plasmid‐derived overexpression of *ompU* (Figure 4a, S3a). Similar survival rates were observed in all strains tested at 0 h. After 2 h of induction of plasmid‐derived *ompU* expression in the Δ*ompU*Δ*rpoE* strain, cell viability significantly decreased compared to the complemented strain Δ*ompU* pBADompU. After 24 h, survival rates of the *ompU*‐expressing double knockout strain dropped significantly, highlighting a disadvantage for the *rpoE* deletion mutant. Strains carrying empty vector controls displayed no significant survival impairment at any of the time points analyzed. Thus, Δ*rpoE* strains are incapable of tolerating high OmpU levels, resulting in severe lethality.

We also tested stress and nonstress conditions using Na‐DC in the growth media. This was done to characterize whether a general lethality phenotype of *ompU* overexpression or an inadequately bile response was exposed. Consistent with Figure 4a, we found a survival deficiency under nonstress conditions upon *ompU* expression (Figure 4b, compare Δ*ompU*Δ*rpoE* mutant with pBAD w/o and pBADompU w/o). In presence of Na‐DC Δ*ompU*Δ*rpoE* pBAD revealed a significant defect at 24 h, consistent with literature (Simonet et al., 2003; Zingl et al., 2020) since OmpU acts as a resistance factor, for example, versus Na‐DC. Furthermore, in presence of Na‐DC OmpU toxicity upon overexpression in Δ*ompU*Δ*rpoE* pBADompU strain, was compensated due to the positive effect of OmpU activity in bile salt resistance. Thus, toxicity of OmpU in Δ*ompU*Δ*rpoE* is a conditional defect as it can be negated upon presence of an antimicrobial compound like bile salts, which required OmpU. Next, we asked whether the severe toxicity of high OmpU levels in Δ*rpoE* cells was related to the inability to produce DegP or other related proteases such as by chance DegS or Tsp (Teoh et al., 2015). Accordingly, *degP* was expressed simultaneously to *ompU* or the respective empty vector control in *trans* in the Δ*ompU*Δ*rpoE* strain. After 2 h of induction of both plasmid‐derived promoter systems, OmpU‐induced lethality of Δ*ompU*Δ*rpoE* cells was significantly reduced compared to the strain exclusively expressing *ompU* (Figures 4c, S3b). However, in similar experiments performed, we found no evidence for lethality reduction in the presence of *degS* or *tsp* encoding plasmids (Figure S3d). We conclude that high OmpU levels in the Δ*rpoE* strain result in severe lethality, which is influenced by the inability to produce periplasmic chaperones/proteases, for example DegP, as this phenotype can be partially rescued by *degP* overexpression.

### 2.5 *ΔrpoE* causes elevated *ompU* expression

**FIGURE 5:**
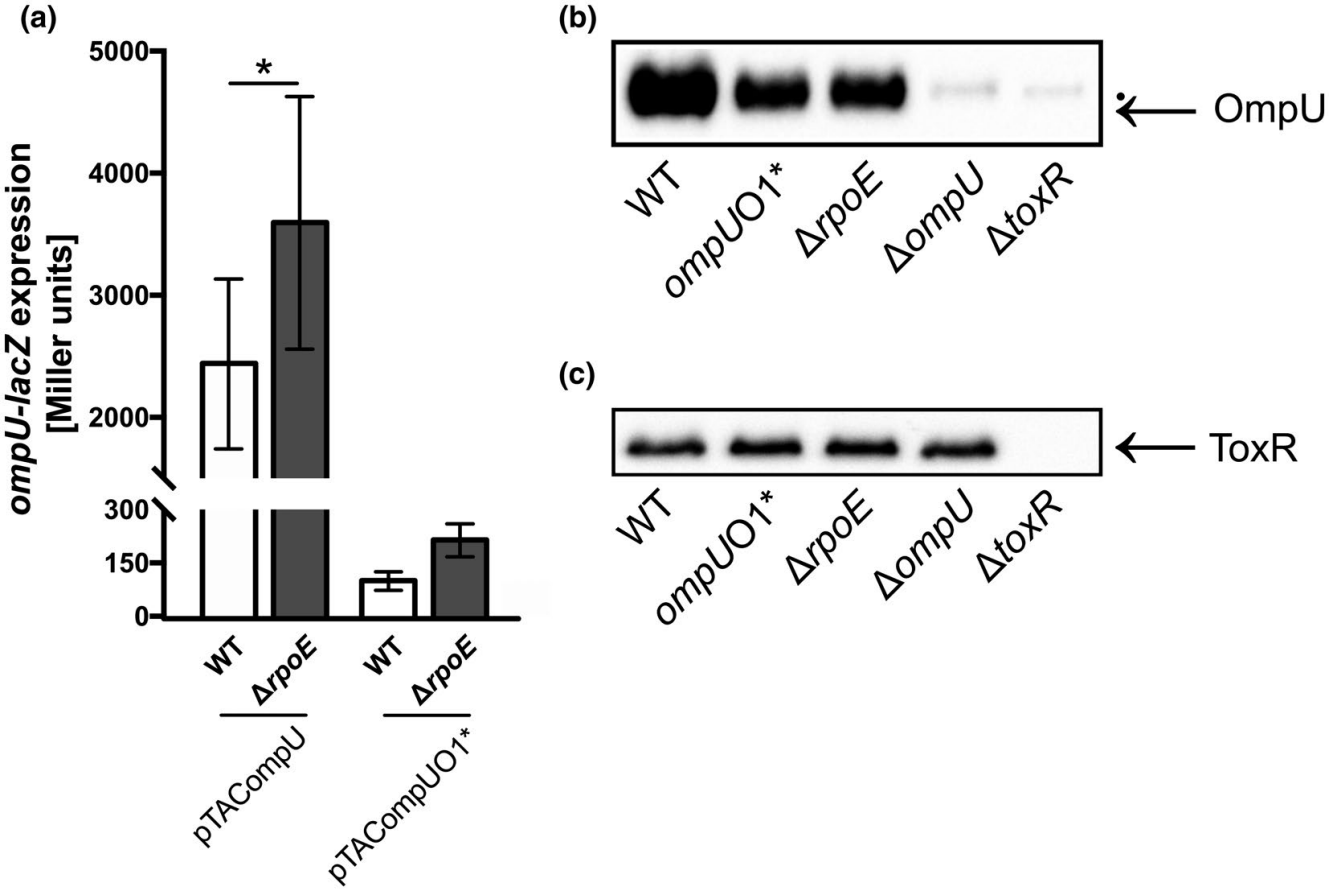
σ^E^‐dependency of *ompU* expression at consistent ToxR levels. (A). β‐galactosidase activities (Miller units) were quantified in WT and Δ*rpoE* harboring pTACompU and pTACompUO1*, respectively, obtained from cultures grown in LB ON. Data were normalized to pTAC, respectively, represent mean values ± standard deviation of six biological replicates and a total sample size of 24. Significant differences between the WT and respective deletion strains are indicated by an asterisk (Student’s *t* test, *p < .05). (b) Immunoblot analysis of WCLs derived from WT, *ompU*O1*, Δ*rpoE*, Δ*ompU* and Δ*toxR* utilizing α‐OmpU antibodies grown in LB ON. (●) represents a nonspecific cross‐reacting background band. (c). Immunoblot analysis of WCLs obtained from WT, *ompU*O1*, Δ*rpoE*, Δ*ompU*, and Δ*toxR* utilizing α‐ToxR antibodies grown in LB ON (For a representative loading and quality control, see Kang‐stained polyacrylamide gel in Figures S4A)

Based on these results, we asked why the Δ*rpoE* strain could not tolerate high OmpU levels and appeared to be unable to regulate *ompU* expression accordingly. To overcome the limitation of *ompU* expression, we aimed to establish a reporter system to quantify and analyze *ompU* expression. Therefore, we cloned the entire *ompU* operator, promoter, and 5′ UTR regions into the promoter‐probe plasmid pTAC3575 encoding for promoterless *lacZ*. To prove differential *ompU* expression regulation in WT and Δ*rpoE* strains, we quantified β‐galactosidase (LacZ) activities (Figure 5a). A significant increase in LacZ activities were observed for pTACompU carrying Δ*rpoE* cells compared to the WT, indicating a σ^E^‐dependent negative transcriptional effect on *ompU* expression. Furthermore, as previously shown, suppressor mutations in the *ompU* promoter control region of Δ*rpoE* cells impair *ompU* promoter‐activated LacZ reporter activity (Davis and Waldor, 2009). In line with these findings, the WT and Δ*rpoE* strain, the latter inherently encodes a *ompU*O1* mutation and carries pTACompUO1* *lacZ*‐reporter plasmid, exhibited a significant decrease in LacZ activities, regardless of the presence or absence of σ^E^. These data indicate that suppressor mutations emerging upon *rpoE* deletion provoke a pronounced downregulation of *ompU* transcription, counteracting the loss of σ^E^‐dependent repression.

To elucidate the amount of OmpU, WCLs of WT, *ompU*O1*, Δ*rpoE*, Δ*ompU*, and Δ*toxR* strains were used and whole cell lysates (WCLs) were prepared. Subsequently, immunoblot analysis using α‐OmpU antibody was performed (Figure 5b). Both strains harboring a single nucleotide deletion in operator O1, that is, *ompU*O1* and cellΔ*rpoE*, displayed decreased OmpU levels compared to the WT (Figures 2and 5b). The observation of reduced OmpU levels in *ompU*O1* strains by immunoblot analysis was in line with the *ompU* expression quantification profile.

In order to exclude the possibility of variable ToxR levels in WT and Δ*rpoE* strains, we also performed immunoblot analysis using α‐ToxR antibody on equal amounts of WCLs (Figures 5a and S4a). Interestingly, ToxR stayed at similar levels displaying minor band intensity variations derived from all tested strains, except for the Δ*toxR* strain, indicating a ToxR‐independent mechanism for downregulation of *ompU*. It is to note that other mechanisms cannot be excluded at this point which may lead to changes of ToxR activity, such as ToxR‐ToxS interaction or changes of disulfide bond formation (Lembke et al., 2020). Additionally, the *toxR* promoter region in Δ*rpoE* cells did not exhibit any mutations (Figure S4b). Hence, these data provide evidence for a σ^E^‐mediated transcriptional mechanism for adjusting *ompU* expression to a physiologically appropriate level.

### 2.6 ompU is autoregulated

**FIGURE 6:**
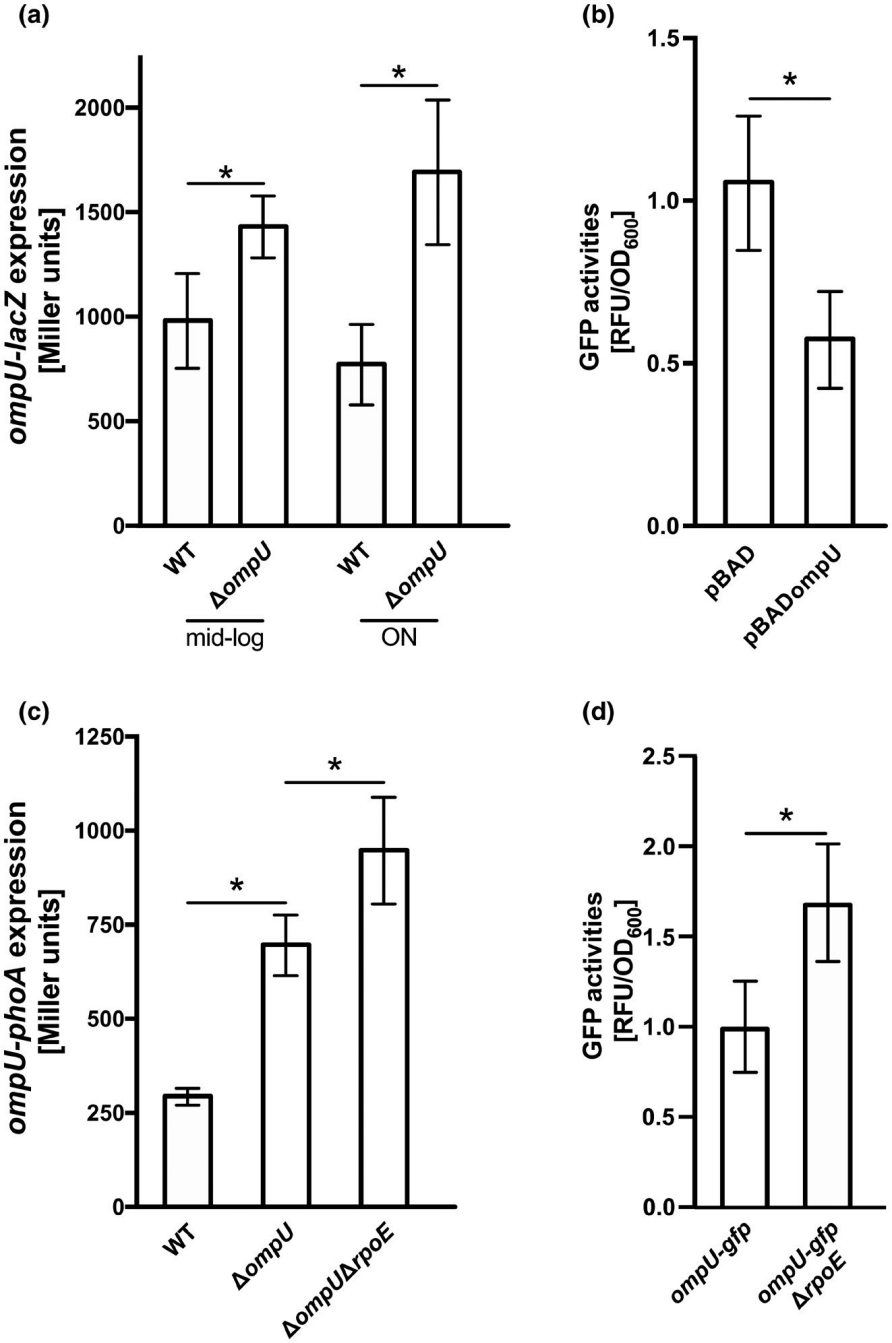
OmpU feedback response on *ompU* expression. (a). β‐galactosidase activities (Miller units) were measured in WT and Δ*ompU* harboring pTACompU grown in LB to mid‐log phase and ON. Data were normalized to pTAC, respectively, represent mean values ± standard deviation of four biological replicates and a total sample size of 8. Significant differences between the WT and respective deletion strains are indicated by an asterisk (one‐way ANOVA followed by a Sidak’s multiple comparisons test, *p < .05). (b). GFP fluorescence quantifications (RFU/OD_600_) were performed in *ompU*‐*gfp* carrying pBAD or pBADompU after 2 h of induction of plasmid‐derived expression with 0.03% arabinose in mid‐log phase in LB. Data represent mean values ± standard deviation of eight biological replicates. Significant differences are indicated by an asterisk (Student’s *t* test, *p < .05). (c). Alkaline phosphatase activities (Miller units) were quantified in WT, Δ*ompU* and Δ*ompU*Δ*rpoE* harboring an *ompU‐phoA* transcriptional fusion grown in LB ON. Data represent mean values ± standard deviation of six biological replicates and a total sample size of 12. Significant differences are indicated by an asterisk (one‐way ANOVA followed by a Sidak’s multiple comparisons test, *p < .05). (d) GFP fluorescence quantifications (RFU/OD_600_) were conducted in *ompU‐gfp* and *ompU‐gfp* Δ*rpoE* incubated overnight in LB. Data represent mean values ± standard deviation of three biological replicates and a total sample size of 9. In the case of *ompU‐gfp* Δ*rpoE*, eight different clones were tested for each three biological and technical replicates were performed, resulting in a total sample size of 72. Significant differences are indicated by an asterisk (Student’s *t* test, *p < .05)

To link the observed impairment of σ^E^ activation of *degP‐phoA* in the Δ*ompU* strain (Figure 3) with the subsequent elevation of *ompU* expression under such conditions (Figure 5a), we attempted to determine whether OmpU had an autoregulative effect on its own expression. To test this hypothesis, we quantified LacZ activities in WT and Δ*ompU* strains carrying pTACompU, reflecting *ompU* promoter activity (Figure 6a). A significant increase in LacZ activity was measured upon deletion of *ompU* compared to WT cells in mid‐log phase, and this difference was even more pronounced after ON incubation.

These results were also confirmed by monitoring *ompU* expression in a GFP fluorescence assay using *ompU*‐*gfp*, in which GFP was translationally fused to the first 15 amino acids of OmpU. There (Figure 6b) overexpression of *ompU* in *trans* resulted in a significant reduction by approximately 50% of relative fluorescence units per OD_600_ (RFU/OD_600_).

Moreover, since Δ*ompU* features a partial deletion of the *ompU* CDS resulting in an OmpU‐deficient strain (Figure 5b), it could still be used for chromosomal *ompU*‐*phoA* fusion. To confirm differential *ompU* regulation in an OmpU‐dependent manner, we measured PhoA activities in *ompU‐phoA* reporter strains harboring a transcriptional fusion of the promoterless *phoA* to residual *ompU* (Figure 6c). In line with the results described above, upon deletion of *ompU*, a high increase in PhoA activity was monitored, which was even greater in a Δ*ompU*Δ*rpoE* background. These findings were further supported by quantifying *ompU* expression utilizing the translational *ompU‐gfp* fusion in an Δ*rpoE* strain (Figure 6d), revealing a significant elevation of RFU/OD_600_ in this deletion background. Taken together, these results highlight the existence of an OmpU‐responsive negative feedback autoregulation of *ompU* expression and additionally provide strong evidence for a σ^E^‐dependent negative effect on *ompU* transcription.

### 2.7 OmpU C‐terminal YDF motif is not an exclusive trigger for σ^E^ activation

**FIGURE 7:**
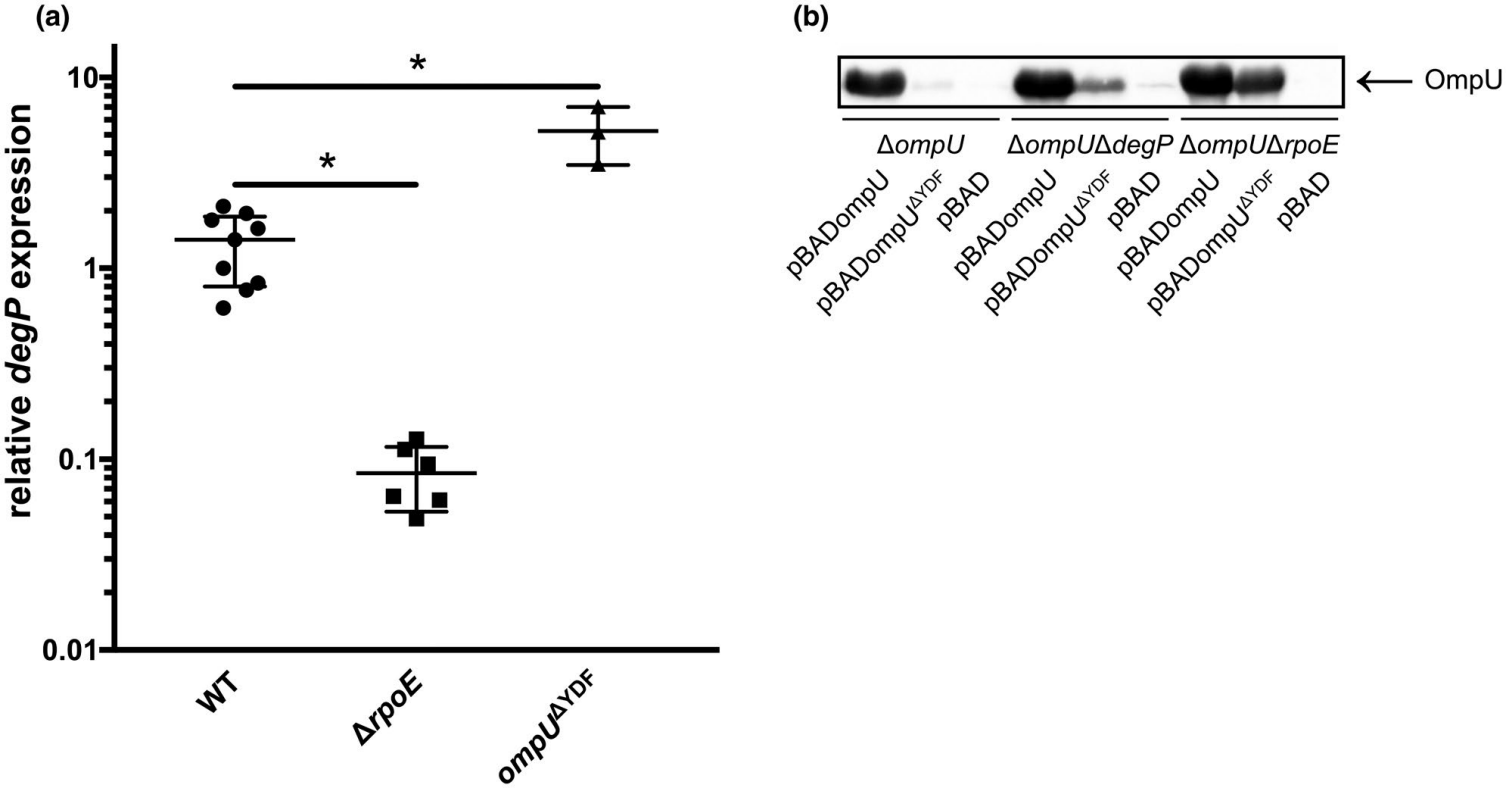
Molecular characterization of OmpU terminal YDF motif. (a) Relative expression levels of *degP* in WT, Δ*rpoE* and *ompU* ^ΔYDF^ were determined by quantitative real time PCR during growth in LB normalized to *rpoB* levels. Data represent mean values ± standard deviation of a minimum of three to nine biological replicates and a total sample size of 9 to 27. Significant differences are indicated by an asterisk (one‐way ANOVA followed by a Dunnett’s multiple comparisons test, *p < .05). (b) Immunoblot analysis of WCLs derived from Δ*ompU*, Δ*ompU*Δ*degP* and Δ*ompU*Δ*rpoE* carrying pBAD, pBADompU or pBADompU^ΔYDF^, respectively, utilizing α‐OmpU antibodies. WCLs were prepared after 2 h of plasmid‐derived expression induction with 0.05% arabinose in LB in mid‐log phase (For a representative loading and quality control, see Kang‐stained polyacrylamide gel in Figures S5)

Previous work indicated that OmpU acts as a sensor for the σ^E^‐mediated periplasmic stress response triggered by antimicrobial peptides, in which the C‐terminal YDF motif of OmpU appears to be the signal that activates the site‐1 protease DegS in *V*. *cholerae* (Mathur et al., 2007). These findings are based on overexpression studies with the 50 C‐terminal amino acids of OmpC fused to cytochrome b_52_, which is anchored to the inner membrane (Mathur and Waldor, 2004; Mathur et al., 2007; Walsh et al., 2003). OmpU is structurally built up as a homotrimer. The C‐terminal YDF motif is localized at the interaction surface between the monomeric subunits buried in the OM (Li et al., 2018). It is unlikely that this signal motif is recognized by a PDZ domain if OmpU is maintained in its intact, folded and assembled state. Besides binding C‐terminal portions of misfolded proteins, PDZ domains are also known to interact with internal peptide sequences (Liu and Fuentes, 2019). *V*. *cholerae* OmpU comprises five YXF motifs, four of which are conserved in *ompU* among other *Vibrio* species (Figure S6). We therefore, revisited the impact of the C’‐terminal located YDF motif and characterized a chromosomal deletion mutant of the C‐terminal YDF motif in order to study its effects on the σ^E^ pathway. We assessed differences in σ^E^ activation by quantifying *degP* mRNA levels in WT, Δ*rpoE*, and *ompU* ^ΔYDF^ strains harvested from cultures grown in LB until mid‐log phase (Figure 7a). The *degP* transcript quantity was 10‐fold reduced in Δ*rpoE* cells, but, unexpectedly, significantly increased in *ompU* ^ΔYDF^ cells compared to the WT. Additionally, OmpU^ΔYDF^ could not be detected by either immunoblot analysis or Kang staining of polyacrylamide gels. To further elucidate whether *ompU* ^ΔYDF^ was insufficiently expressed or served as target of rapid proteolysis, we overexpressed either *ompU* or *ompU* ^ΔYDF^ in Δ*ompU*, Δ*ompU*Δ*degP*, and Δ*ompU*Δ*rpoE* strains and prepared WCLs for SDS‐PAGE and immunoblot analysis using α‐OmpU antibodies. OmpU was detected in all strains tested. OmpU^ΔYDF^ remained undetectable in Δ*ompU* cells, whereas low amounts were detected in Δ*ompU*Δ*degP*, and higher OmpU levels in the Δ*ompU*Δ*rpoE* strain as observe in Kang‐stained SDS‐PAGE and immunoblot (Figures S5 and 7b). These data suggest that OmpU^ΔYDF^ may occur in an unstable state, and thereby activate σ^E^ release, resulting in high protease production and activation rates, for example of *degP*.

## 3 DISCUSSION

The transition of *V*. *cholerae* from an aquatic environment to the human host requires a quick change in its expression profile. Adaption to antimicrobial effectors, for example bile salts, is crucial for the pathogen to fully activate colonization and virulence factor expression. This process involves changes in the OM profile, including the removal of OmpT by outer membrane vesicle production, as well as by transcriptional repression, and the simultaneous activation of OmpU biosynthesis (Provenzano and Klose, 2000; Zingl et al., 2020). This inversion of OMP production is ensured by activation of the main virulence regulator ToxR upon host entry, for example by bile salts (Lembke et al., 2018). ToxR recognizes AT‐rich DNA elements located upstream of a particular CDS with its winged helix‐turn‐helix motif (Goss et al., 2013; Morgan et al., 2019). These specific ToxR boxes are each separated by five base pairs. Moreover, σ^E^ is known to be essential for stress responses in *E*. *coli* and other Gram‐negative bacteria, and disruption of *rpoE* leads to suppressor mutations (Button et al., 2007; De Las Penas et al., 1997; Heusipp et al., 2003). Studies of σ^E^ in *V*. *cholerae* show its requirement for intestinal colonization, in RIP of ToxR, as well as in cell wall damage, which may be provoked by antimicrobial peptides or β‐lactam antibiotics (Almagro‐Moreno et al., 2015; Ding et al., 2004; Kovacikova and Skorupski, 2002; Mathur et al., 2007; Weaver et al., 2018). Several different suppressor mutations within the 5′ upstream regulatory and noncoding regions of *ompU* were identified upon *rpoE* depletion, causing reduced OmpU levels (Davis and Waldor, 2009).

In this work, we characterized the survival ability of *V*. *cholerae* with respect to the σ^E^ pathway and σ^E^‐controlled *ompU* expression. We present comprehensive data on the relationship between the σ^E^ pathway, suppressor genotypes in *rpoE* knockout mutants, and the expression status of *ompU*. The Δ*rpoE* strains analyzed herein exhibited single nucleotide deletions or insertions within the five base pairs stated, separating the two ToxR boxes of an operator, in turn leading to decreased *ompU* expression, similar to what has been reported earlier (Davis and Waldor, 2009). Remarkably, comparable results were obtained for the Δ*degS* strain, which is deficient in the site‐1 protease responsible for RIP of the anti‐sigma factor complex RseA, and liberation of σ^E^ (Kim, 2015). This indicates that either the lack of *rpoE* or the inability to liberate σ^E^ from RseA leads to segregation of similar suppressor mutations in the upstream region of *ompU*, causing a downregulation of *ompU* expression. In silico analysis of the classical strain O395 revealed an intact *rpoE* sequence and a similar organized *ompU* upstream region. Interestingly, the spacer region between ToxR boxes O1 and O2 harbors one additional adenine, similar as found herein and by Davis and Waldor (2009) in El Tor *rpoE* knockout backgrounds. In our opinion, this finding may not indicate evidence for a correlation between *rpoE* mutants and changed *ompU* upstream regions per se, and deeper analysis of expression studies of *ompU*, in combinations with *rpoE* knockout mutants would be needed for clarification for the classical strain. For the in herein characterized Δ*ompU* ElTor strain the role of OmpU itself was characterized, revealing that *rpoE* depletion in Δ*ompU* strains was obtained without any mutations in the upstream *ompU* region. Since the deletion mutations described above were identified in ToxR box O1, it seems likely that this may have caused a conflict, resulting in inefficient DNA accessibility for ToxR or ToxR‐ToxR interaction (Lembke et al., 2020), and eventually reduced transcription initiation of *ompU*.

Additional data show, as discussed below, that suppressor mutations in the *ompU* expression control region were selected for rescue of cell growth, as the levels of OmpU in Δ*rpoE* strains play a critical role. As established earlier, the *ompU* promoter control region consists of three ToxR binding sites, O1‐O3, each separated by a spacer region (Goss et al., 2013; Morgan et al., 2019). O3 is located immediately upstream of the core promoter, followed by O2 and O1, respectively. Comprehensive analysis of a single base pair deletion within and complete removal of the entire spacer region from O1 to O3 revealed successive loss of *ompU* expression strength. Therefore, the spacer region of operator O3 was identified to be essential. Interestingly, bile salt‐dependent activation of *ompU* expression was activated in a Δ*rpoE* background, as well as in the O1* and O2* constructed strains. In contrast, deletions of the respective spacer regions always resulted in a decrease in bile‐dependent *ompU* expression. Therefore, we conclude that by genetic evidence all three operator regions are important in order to fully respond to bile salts and disabled operator‐ToxR interaction is compensated in O1* and O2* strains. Interestingly, we recently published that in a bacterial two hybrid system ToxR‐ToxR interactions have the highest activity in the presence of operator sites and bile salts (Lembke et al., 2020). This may indicate that bile influences ToxR interaction in a way to better fit to operators even if the spacing is reduced. Furthermore, if bile salts are present, *V*. *cholerae* cells tolerate higher levels of *ompU* expression, even in a Δ*rpoE* deletion background. These data suggest that another physiological response is activated in the presence of bile salts, providing high levels of OmpU in WT and Δ*rpoE* strains, not leading to a growth conflict.

The above discussed results indicate that *ompU* expression itself causes a condition in favor of selection of suppressor mutations in strains lacking *rpoE* or *degS*. Therefore, we characterized the impact of *ompU* expression and its role in the σ^E^ pathway response by monitoring the expression of *degP*. Obtained data showed a strong decrease in *degP* expression in Δ*rpoE*, Δ*ompU*, and Δ*ompU*Δ*rpoE* strains. Functional complementation reversed this phenotype in both single deletion strains, whereas *ompU* complementation in the double knockout strain had no effect on *degP* expression. Thus, these data indicate that while OmpU positively controls σ^E^ activity, which in turn acts negatively on *ompU* expression, yields in the conclusion that deletion of *rpoE* or *ompU* acts negative on *degP* expression control. Additionally, our data confirm and identify OmpU as a trigger for σ^E^ pathway activation, as suggested previously (Mathur et al., 2007).

Further experiments addressed the role of OmpU in growth and survival. Overexpression of *ompU* was associated with growth defects in *rpoE‐*deficient strains. Moreover, toxicity of the *ompU* overexpression in Δ*ompU*Δ*rpoE* is negated in presence of bile/Na‐DC. Interestingly, survival deficiency was rescued by DegP, indicating that loss of DegP‐controlled OmpU synthesis or assembly leads to harmful conditions in the periplasm or OM. Hence, it is tempting to speculate whether a σ^E^‐controlled downstream factor, for example DegP, is involved in the feedback regulation of OmpU.

Such feedback response adjusts the regulation of *ompU* expression according to cell stress and survival function. Thereby, we asked whether this control mechanism links *ompU* expression to the σ^E^ pathway. The underlying mechanism may involve factors that are under σ^E^ control acting negatively on *ompU* expression. Based on the fact that Δ*rpoE* strains exhibited suppressor mutations causing *ompU* downregulation, characterization of *ompU* expression in the *rpoE* knockout strain was limited. Therefore, we reconstructed one suppressor mutation (O1*) on the chromosome in a *rpoE^+^* background. Consistent with the original *rpoE* suppressor mutant, the reconstructed strain (O1*) identified this mutation as an *ompU* promoter‐down mutation exhibiting lower OmpU protein levels in an *rpoE^+^* background. No *toxR* mutation or change in ToxR levels was observed in these strains; therefore, we found no evidence for any involvement of ToxR. Furthermore, a plasmid‐based quantification method was used to measure *ompU* transcription under WT and O1* promoter activity in a promoter‐probe plasmid in WT and *rpoE* knockout strains (Atlung et al., 1991). The results showed that WT *ompU* promoter activity tested in the *rpoE* knockout strain was accompanied by a strong increase in *ompU* expression, whereas the O1* promoter mutation caused its downregulation, independent of *rpoE*. These data strongly suggest the existence of a yet unknown negative feedback regulation circuit between the σ^E^ pathway and *ompU* expression. Recently, σ^E^‐dependent expression of the small inhibitory regulative RNA MicV was found to affect *ompU* expression (Peschek et al., 2019). However, a *micV* knockout mutant did not interfere with *ompU* expression under the conditions tested (data not shown), suggesting an involvement of additional components.

To gain evidence in support of the involvement of OmpU itself within a negative regulatory pathway, we tested transcriptional and translational reporter fusions in WT and Δ*ompU* strains. For all reporter systems used, we identified autoregulatory effects, showing increased *ompU* promoter activity for the plasmid‐encoded *ompU* promoter‐*lacZ* fusion, and for chromosomally encoded *ompU‐phoA* reporter systems in the *ompU^‐^* background. Additionally, similar effects were observed in an *ompU‐gfp* reporter strain upon *ompU* overexpression. Hence, OmpU also has a negative effect on its own expression, operating in a negative feedback mode.

Since OmpU seems central to the activation of the σ^E^ system, a more detailed characterization of OmpU and its role as a sensor was performed. Our findings indicate that DegS’s PDZ domain also recognizes internal YXF motifs of OmpU, eventually resulting in σ^E^ release, and causing elevated *degP* expression in *ompU* ^ΔYDF^ cells compared to the WT. Moreover, *degP* is under σ^E^ control, comprises PDZ domains, and is involved in the periplasmic stress response by acting either as a multimeric chaperone or protease on misfolded periplasmic proteins, for example OmpU (Kolmar et al., 1996, Krojer et al., 2008). Plasmid‐derived OmpU^ΔYDF^ protein was hardly detectable in Δ*ompU* cells. Remarkably, OmpU^ΔYDF^ was regained successively in the Δ*degP* and Δ*rpoE* strains, respectively, indicating σ^E^‐dependent proteolysis, for example by DegP.

**FIGURE 8:**
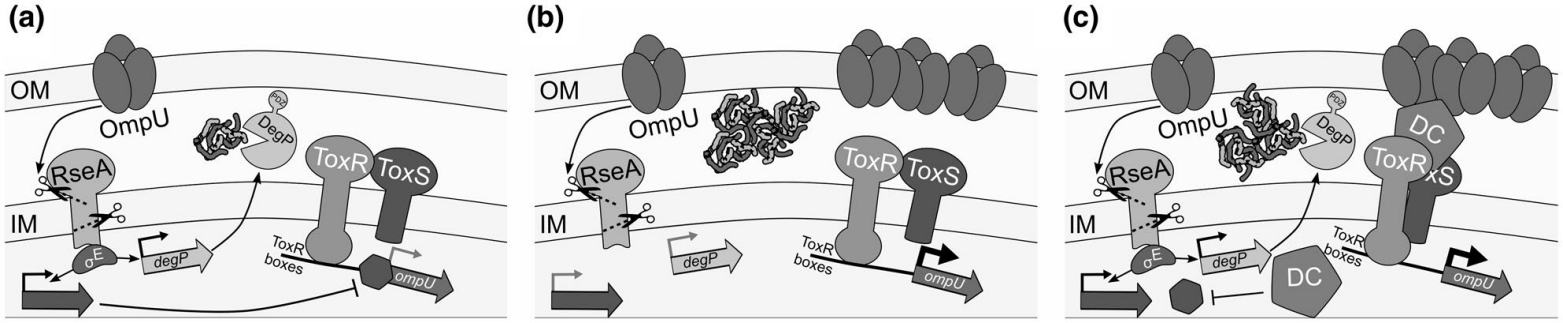
A regulatory circuit indicates the interplay between the major outer membrane porin OmpU, the σ^E^ response pathway and periplasmic stress stimuli in *V*.*cholerae*. This study reveals a negative feedback regulation resulting in fine‐tuning of the *ompU* expression level, balancing stress response and survival. In (a), OmpU serves to trigger DegS proteolytic attack (first scissor in the periplasm scheme) at the anti‐sigma factor RseA followed by attack through RseP (second scissor intra membrane scheme), which in consequence lead to a release of RpoE/ σ^E^ to act on gene regulation, for example, *degP* and others not shown. Also shown as dark gray arrow, a yet unknown regulatory factor mediate negative regulation on *ompU* expression. (b) in case of *rpoE* knockout mutation no negative regulation takes place, subsequently *ompU* promoter activity increases, as shown herein. As a consequence, *rpoE* deletion mutants can only be obtained if point mutations arise which decrease *ompU* expression. (c) for WT the presence of bile leads to increased *ompU* expression, either indicating bona fide ToxRS activation, or through inhibition of RpoE negative feedback regulation. Whether bile activation mechanism is separated from RpoE pathway or acts in concert with ToxRS must be further characterized

In summary, the present work states multiple levels of OmpU regulation that ultimately influence the effectiveness of *V*. *cholerae* in establishing a σ^E^ pathway response. The ability to modulate the OM, and eventually the presence of OmpU in the OM, affects its colonization fitness, bile resistance, and intestinal survival. Therefore, fine‐adjusted orchestration of properly folded OmpU under unstressed conditions, accurate expression of *ompU* at a physiologically relevant level, and feedback responses to membrane stress are highlighted by various experiments reported herein. It is appealing to speculate that OmpU levels and folding states are crucial for proper physiological counteraction of membrane stress conditions. The results obtained in this study point to the possibility of the existence of a σ^E^‐regulated repressor element that is responsible for transcriptional downregulation of *ompU*, as suggested by our model (Figure 8). Thus, negative feedback regulation of *ompU* expression and stability by σ^E^‐dependent factors could represent a fine‐tuned mechanism on multiple levels relevant for proper OmpU function and modulation of the signal transducer for membrane stress.

## 4 EXPERIMENTAL PROCEDURES

### 4.1 Bacteria, plasmids and growth conditions

Bacterial strains and plasmids used in this study are listed in Table 1; oligonucleotides are listed in Table 2. *V*. *cholerae* P27459‐S, a spontaneous streptomycin (Sm)‐resistant mutant of the clinical isolate O1 El Tor was used as wild type (WT) (Pearson et al., 1993). *Escherichia coli* strains DH5αλ*pir* and SM10λ*pir* were used for genetic manipulation (Hanahan, 1983; Kolter et al., 1978; Miller and Mekalanos, 1988). The latter strain was used to deliver plasmids to *V*. *cholerae* by conjugation. Unless stated otherwise, strains were cultured in lysogeny broth (LB) (1% tryptone, 1% NaCl, and 0.5% yeast extract) either shaking with 180 rpm or in agar plates with aeration at 37°C. *V*. *cholerae* was usually incubated overnight (ON) or inoculated with a starting OD_600_ of 0.1 and grown to mid‐log phase followed by the induction of plasmid‐derived expression. If required, antibiotics or other supplements were used in the following final concentrations: streptomycin (Sm) 100 µg/ml, kanamycin (Km) 50 µg/ml, chloramphenicol (Cm) 2 or 5 µg/ml, ampicillin (Ap) 100 µg/ml or 50 µg/ml in combination with other antibiotics, D‐glucose (Gluc) 0.2%, L‐arabinose (Ara) 0.05%, sodium‐deoxycholate (Na‐DC, Sigma‐Aldrich) 0.1%.

**TABLE 1:** Strains and plasmids used in this study

Strain or plasmid	Genotype, description, resistance	References
*E*. *coli*		
DH5αλ*pir*	F^‐^Φ80Δ*lacZ*Δ*M15*Δ(*argF lac*)*U169 deoR recA1 endA1 hsdR17* (r_K_^‐^m_K_^+^) *supE44 thi‐1 gyrA69 relA1*, λ*pir*R6K	Hanahan (1983)
SM10λ*pir*	*thi thr leu tonA lacY supE recA*::RP4‐2‐Tc:Mu λ*pir*R6K, Km^r^	Miller and Mekalanos (1988)
*V*. *cholerae*		
WT	P27459‐S, O1 Inaba, El Tor, clinical isolate, Bangladesh 1976, spontaneously Sm^r^	Pearson et al. (1993)
Δ*toxR*	P27459‐S Δ*toxR*, deletion in *toxR*, Sm^r^	Fengler et al. (2012)
Δ*ompU*	P27459‐S Δ*ompU*, 825 bp in‐frame deletion in *ompU*, Sm^r^	Provenzano and Klose (2000)
Δ*degS*	P27459‐S Δ*degS*::*cat*, *degS* replaced by *cat* cassette, single nucleotide deletion in *ompU* operator O1, Sm^r^, Cm^r^	Lembke et al. (2018)
Δ*degP*	P27459‐S Δ*degP*::*cat*, *degP* replaced by *cat* cassette , Sm^r^	Lembke et al. (2018)
Δ*rpoE*	P27459‐S Δ*rpoE*::*cat*, *rpoE* replaced by *cat* cassette, single nucleotide deletion in *ompU* operator O1 ompUO1*, Sm^r^, Cm^r^	This study
Δ*ompU* Δ*rpoE*	P27459‐S Δ*ompU* ΔrpoE::*cat*, *rpoE* replaced by *cat* cassette in Δ*ompU* background, Sm^r^, Cm^r^	This study
*ompU* ^ΔYDF^	P27459‐S *ompU*ΔYDF, in‐frame deletion of the C‐terminal amino acids Tyr, Asp, Phe (AA position 348 ‐ 350) of *ompU*, Sm^r^	This study
*ompU*∆O1	P27459‐S *ompU*∆O1, deletion of *ompU* operator O1, Sm^r^	This study
*ompU*O1*	P27459‐S *ompU*O1*, single nucleotide deletion between the two ToxR boxes in *ompU* operator O1, Sm^r^	This study
*ompU*∆O2	P27459‐S *ompU*∆O2, deletion of *ompU* operator O2, Sm^r^	This study
*ompU*O2*	P27459‐S *ompU*O2*, single nucleotide deletion between the two ToxR boxes in *ompU* operator O2, Sm^r^	This study
*ompU*∆O3	P27459‐S *ompU*∆O3, deletion of *ompU* operator O3, Sm^r^	This study
*ompU*O3*	P27459‐S *ompU*O3*, single nucleotide deletion between the two ToxR boxes in *ompU* operator O3, Sm^r^	This study
*ompU*‐*gfp*	P27459‐S *ompU*‐*gfp*, 15 N‐terminal AA of *ompU* fused in frame to *gfp*, *ompU^‐^* Sm^r^	This study
*ompU*‐*gfp* Δ*rpoE*	P27459‐S *ompU*‐*gfp* Δ*rpoE*, *rpoE* replaced by *cat* cassette in *ompU‐gfp* background, 15 N‐terminal AA of *ompU* fused in frame to *gfp*, *ompU^‐^* Sm^r^, Cm^r^	This study
WT *degP‐phoA*	P27459‐S with insertion of pGP704phoA downstream of *degP*, Sm^r^, Ap^r^	Lembke et al. (2018)
Δ*ompU degP*‐*phoA*	P27459‐S Δ*ompU* with insertion of pGP704phoA downstream of *degP*, Sm^r^, Ap^r^,	This study
Δ*rpoE*::*cat degP*‐*phoA*	P27459‐S Δ*rpoE*::*cat* with insertion of pGP704phoA downstream of *degP*, Sm^r^, Ap^r^, Cm^r^	This study
Δ*ompU* Δ*rpoE*::*cat degP*‐*phoA*	P27459‐S Δ*ompU* Δ*rpoE*::*cat* with insertion of pGP704phoA downstream of *degP*, Sm^r^, Ap^r^, Cm^r^	This study
Δ*ompU ompU‐phoA*	P27459‐S Δ*ompU* with insertion of pGP704phoA downstream of *ompU*, Sm^r^, Ap^r^	This study
Δ*ompU* Δ*rpoE*::*cat ompU‐phoA*	P27459‐S Δ*ompU* Δ*rpoE*::*cat* with insertion of pGP704phoA downstream of *ompU*, Sm^r^, Ap^r^, Cm^r^	This study
Plasmids		
pCVD442	Suicide vector, *ori_R6K_*, *mobRP4*, *sacB*, Ap^r^	Donnenberg and Kaper (1991)
pBAD18‐Kanbla^‐^	Expression vector, *ori_ColE1_*, arabinose inducible, deletion of *bla* sequence, Km^r^	Guzman et al. (1995)
pMMB67EH	Expression vector, RSF1010 *oriV*, RSF1010 *oriT*, *lacI*, RSF1010 *repABC*, IPTG inducible, Ap^r^	Morales et al. (1991)
pACYC184	Cloning vector, *ori_p15A_*, Tet^r^, Cm^r^	Rose (1988)
pGP704phoA	Cloning vector, *ori_R6K_*, *mobRP4*, promoterless *phoA* of SM10λ*pir*, Ap^r^	Berg et al. (2007)
pTAC3575	pBR322 ori, promoterless *phoA* and *lacZ*, Ap^r^	Atlung et al. (1991)
pCVD442rpoE::cat	pCVD442 encoding for up and down fragments of *rpoE* from P27459‐S flanking a *cat* cassette, Ap^r^, Cm^r^	This study
pCVD442ompUΔO1	pCVD442 encoding for up and down fragments of *ompU* O1 from P27459‐S, Ap^r^	This study
pCVD442ompUO1*	pCVD442 encoding for up and down fragments of *ompU* O1 from P27459‐S Δ*rpoE*::*cat* to achieve a single nucleotide deletion between the two respective ToxR boxes, Ap^r^	This study
pCVD442ompUΔO2	pCVD442 encoding for up and down fragments of *ompU* O2 from P27459‐S, Ap^r^	This study
pCVD442ompUO2*	pCVD442 encoding for up and down fragments of *ompU* O2 from P27459‐S to achieve a single nucleotide deletion between the two respective ToxR boxes, Ap^r^	This study
pCVD442ompUΔO3	pCVD442 encoding for up and down fragments of *ompU* O3 from P27459‐S, Ap^r^	This study
pCVD442ompUO3*	pCVD442 encoding for up and down fragments of *ompU* O3 from P27459‐S to achieve a single nucleotide deletion between the two respective ToxR boxes, Ap^r^	This study
pCVD442ompUΔYDF	pCVD442 encoding for up and down fragments of *ompU* from P27459‐S to achieve a deletion of the C‐terminal AA Tyr, Asp and Phe (AA position 348–350)	This study
pCVD442ompU‐gfp	pCVD442 encoding for up and down fragments of *ompU* from P27459‐S comprising the *ompU* 5′UTR, RBS and the first 15 AA fused in frame to *gfp* to achieve a translational fusion	This study
pBAD18‐Kanbla^‐^ rpoE‐FLAGrseABC	*rpoErseABC* cloned from P27459‐S on pBAD18‐Kanbla^‐^ with an in‐frame FLAG‐tag fused to C‐terminal *rpoE*, Km^r^	This study
pBAD18‐Kanbla^‐^ompU	*ompU* from P27459‐S on pBAD18‐Kanbla^‐^, Km^r^	This study
pMMB67EHdegP	*degP* of P27459‐S in pMMB67EH, Ap^r^	Lembke et al. (2018)
pGPdegPphoA	pGP704phoA with *degP* gene fragment from P27459‐S, Ap^r^	Lembke et al. (2018)
pGPompUphoA	pGP704phoA with *ompU* gene fragment from P27459‐S, Ap^r^	Lembke et al. (2018)
pTAC3575ompU	Operator and promoter region of *ompU* from P27459‐S cloned in the same orientation as promoterless *lacZ* on pTAC3575, Ap^r^	This study
pTAC3575ompUO1*	Operator and promoter region of *ompU* harboring a single nucleotide deletion in operator O1 from P27459‐S Δ*rpoE*::*cat* cloned in the same orientation as promoterless *lacZ* on pTAC3575, Ap^r^	This study

**TABLE 2:** Oligonucleotides used in this study

Primer name	Sequence (5′ to 3′)^†^
F1_rpoE‐KO_*Xba*I_fw	ATATCTAGAGCGGCCCTTTGCTTAATA
F1_rpoE‐KO_*Hind*III_rv	ATAAAGCTTTCGAGCGGTCACTCCTAT
cat‐pACYC184_*Hind*III_fw	ATAAAG CTTAGCACCTCAAAAACACCATC
cat‐pACYC184_*Bam*HI_rv	TTAGGATCCCACCAGGCGTTTAAGGGCA
F2_rpoE‐KO_*Bam*HI_fw	ATAGGATCCCGCAAATTCCGTAATGAC
F2_rpoE‐KO_*Sac*I_rv	ATAGAGCTCTCATTCCACAACCGATTC
F1_ompU‐KO_*Sac*I_fw	ATAGAGCTCAAATCTTTATGCTTTATGGTTGAA
F1_ompU‐KO_*Nde*I_rv	AATCATATGATTAAGTCCTAATTTA
F2_ompU‐KO_*Nde*I_fw	AATCATATGGCGTTTACTGCGACAT
F2_ompU‐KO_*Sph*I_rv	ATAGCATGCAGTATAACCGAGATAGAAAAACGCCT
F1_ompU_*Xba*I_fw	AATCTAGAACAAAGGCCAATTTGGTG
F2_ompU_*Sac*I_rev	ATTGAGCTCATCTGCGGTGGTCAG
F1_ompUΔYDF_SOE_rv	**CAATTA**ACGTAGACCGATAGCCAG
F2_ompUΔYDF_SOE_fw	**CTACGT**TAATTGTTGACTTCAGGTCAC
F1_ompU‐op_*Xba*I_fw	AATTCTAGATGGTATTCCGCATTCTCTTTC
F2_ompU‐op_*Sac*I_rv	AATGAGCTCTTGAACCTGCTTTGTCACCGC
F1_ompU‐ΔO1_SOE_rv	TGTTGTATTTCGAGATTGAGCAAAATGCACGCAATC
F2_ompU‐ΔO1_SOE_fw	CAATCTCGAAATACAACAAATTAAATTAAAAAAAACACTTAC
F1_ompU‐ΔO2_SOE_rv	AGTTATTGTATGTTTGTTGGTAAGTGTTTTTTTTAA
F2_ompU‐ΔO2_SOE_fw	AACAAACATACAATAACTTGATAAATTTTTACCAAC
F1_ompU‐O2*_SOE_rv	GTTTTTAAAGATTTTAATGTTTGTTGGTAAGTGTTT
F2_ompU‐O2*_SOE_fw	ATTAAAATCTTTAAAAACAATAACTTGATAAATTTT
F1_ompU‐op_*Sac*I_fw	AATGAGCTCTGGTATTCCGCATTCTCTTTC
F2_ompU‐op_*Sph*I_rv	AATGCATGCCAGAGTACTTGGCAGCGT
F1_ompU‐ΔO3_SOE_rv	ATCAGTTAGTCCAACTTATGAACACTGTTTTATTGT
F2_ompU‐ΔO3_SOE_fw	TAAGTTGGACTAACTGATAGCGGAACTTTGGGAGTA
F1_ompU‐O3*_SOE_rv	AGTAAAATGTATAAATCCAACTTATGAACACTGTTT
F2_ompU‐O3*_SOE_fw	GGATTTATACATTTTACTAACTGATAGCGGAACTTT
F1_ompU‐gfp_*Xba*I_fw	ATTTCTAGACATCATTTACCTCTGCGCCAAAG
F1_ompU‐gfp_SOE_rv	CTTTACTCACTGCAGCAGCTGATACAG
F2_ompU‐gfp_SOE_fw	GCAGTGAGTAAAGGAGAAGAACTTTTCACTG
F2_ompU‐gfp_SOE_rv	TCAACAACTATTTGTATAGTTCATCCATGCC
F3_ompU‐gfp_SOE_fw	CAAATAGTTGTTGACTTCAGGTCACACG
F3_ompU‐gfp_*Sac*I_rv	ATTGAGCTCGTCACGCTGATGGCTTGC
rpoE‐FLAG_*Kpn*I_fw	ATAGGTACCGAAATTGTCGAAAAATTC
rpoE‐FLAG_*Xba*I_rv	ATATCTAGATTACTTGTCATCGTCGTCCTTGTAGTCCAGAAGAGGTTTGATTTTCTT
ompU_*Sac*I_fw	AATGAGCTCCGTGGCTTACGTCGCACA
ompU_*Sph*I_rv	AATGCATGCTTAGAAGTCGTAACGTAG
ompU‐prom_*Bam*HI_fw	ATTGGATCCTTACCTCTGCGCCAAAGT
ompU‐prom_*Xba*I_rv	ATTTCTAGATTTGTGCGACGTAAGCCA
rpoB_fw	CTG TCT CAA GCC GGT TAC AA
rpoB_rv	TTT CTA CCA GTG CAG AGA TGC
degP_fw	GCT TCC TCT CTC AGT CAA T
degP_rv	CAG ACG CTG TCT TGA AAC T

^†^ Restriction sites are underlined; site directed mutations are in bold.

### 4.2 Construction of in‐frame mutants, translational gfp fusions, expression plasmids and promoter fusions

The isolation of chromosomal DNA, plasmids or PCR products, and the construction of chromosomal transcriptional *phoA* fusions, constructs and in‐frame deletion mutants were carried out as described previously (Seper et al., 2011). Plasmids, digested plasmids and PCR products were purified using the QIAquick^®^ PCR purification, the QIAquick^®^ gel extraction, or the QIAprep^®^ Spin Mini Kit (Qiagen). PCRs for subcloning and sequencing were performed using Q5^®^ DNA polymerase (New England Biolabs); for all other PCRs *Taq* DNA polymerase (New England Biolabs) was used.

Deletion strains were constructed by using derivatives of the suicide vector pCVD442 (Donnenberg and Kaper, 1991). Start to stop deletion of *rpoE*, *ompU* operator point mutations, *ompU* operator space region deletions (Figure S1), *ompU* truncations and translational *gfp* fusions in *V*. *cholerae* P27459‐S were generated via SOE‐PCR (splicing by overlap extension, (Horton et al., 1989)). A *cat* cassette from pACYC184 was obtained to improve the selection of *rpoE* deletion strains. The generation of overlapping regions of two PCR fragments allowed the annealing of the two PCR products in another PCR. Respective suicide vectors were generated by PCR amplification of 600–1,000 bp up‐ and downstream of the locus of interest using the corresponding oligonucleotide pairs (Table 2). The resulting fragments were digested with the appropriate restriction enzyme indicated by the name of the oligonucleotide, and finally ligated into the identically digested suicide vector backbone of pCVD442. Subsequently, the cloned suicide plasmids were first transferred into electro competent *E*. *coli* DH5αλ*pir*. Positive clones were selected by colony PCR and Sanger‐sequencing (data not shown) and afterward transferred into electro competent SM10λ*pir* in order to transfer the construct to *V*. *cholerae* by conjugation. Cells were grown on agar plates containing Sm and Ap to select for clones which integrated the deletion construct into the chromosome by homologous recombination, followed by the incubation on sucrose (10%) agar plates to obtain Ap^S^ clones in which in turn an excision of the suicide vector from the chromosome occurred. Correct gene deletions were confirmed by colony PCR and Sanger‐sequencing (data not shown).

pBAD18‐Kanbla^‐^ expression constructs were generated by PCR or SOE‐PCR of the respective coding region including a ribosome binding site (RBS). The resulting fragments were digested with the appropriate restriction enzyme indicated by the name of the oligonucleotide (Table 2) and eventually ligated into the identically digested pBAD18‐Kanbla^‐^ vector backbone. The obtained constructs were transferred into electro competent DH5αλ*pir* and Km^R^ colonies were screened by colony PCR for the existing cloned constructs which were additionally verified by Sanger‐sequencing and by immunoblotting for correct expression (data not shown). Subsequently, plasmids were isolated and transferred by electroporation into *V*. *cholerae* strains, which were also tested for expression by immunoblotting before being used in other experiments like survival plating and the analysis of membrane stress response.

Derivatives of pTAC3575 were constructed to obtain plasmid‐encoded transcriptional fusions of *lacZ* to respective operator and/or promoter regions. Respective noncoding regions of *ompU* and *degP*, including the annotated transcriptional start site and parts of the 5′UTR, were amplified by PCR or SOE‐PCR using the appropriate oligonucleotide pairs (Table 2). Amplicons were digested by *Bam*HI and *Xba*I, ligated into identically digested pTAC3575, transferred by electroporation into DH5αλ*pir* and Ap^R^ colonies were determined by colony PCR for the existing cloned constructs which were additionally verified by Sanger‐sequencing (data not shown). Subsequently, plasmids were isolated and transferred by electroporation into *V*. *cholerae* strains, which were used for the determination of β‐galactosidase activity depending on the power of the cloned promoters in the respective deletion strains.

### 4.3 Alkaline phosphatase (PhoA) assay

To determine the enzymatic activities of transcriptional *phoA* fusions, alkaline phosphatase assays were performed as described previously (Miller, 1992). Respective *V*. *cholerae* strains were grown in LB at 37°C, 180 rpm, ON. Cell disruption was performed with sodium dodecyl sulfate (SDS) and chloroform. Experiments were performed with a minimum of six biological replicates and technical duplicates. PhoA activities were expressed in Miller units, calculated as following: MU = (OD_405_ × 1000) / (OD_600_ × t_min_ × *v*_ml_ × 0.96).

### 4.4 β‐galactosidase (LacZ) assay

To determine the enzymatic activities of transcriptional *lacZ* fusions, β‐galactosidase assays were performed as described previously (Miller, 1992). Respective *V*. *cholerae* strains were grown in LB at 37°C, 180 rpm, ON. Cell disruption was performed with SDS and chloroform. Experiments were performed with a minimum of six biological replicates and technical duplicates. LacZ activities were expressed in Miller units, calculated as following: MU = (OD_405_ × 1000) / (OD_600_ × *t*_min_ × *v*_ml_ × 0.486).

### 4.5 Analysis of ompU translation by GFP quantification

To quantify *ompU* translation in *V*. *cholerae*, respective *gfp* fusion strains were incubated in LB at 37°C, 180 rpm, ON, cultures were pelleted and 1 OD_600_ unit was resuspended in 1 ml saline. 1:10 dilutions were measured in 96 well fluorescence plates (Costar) using the FLUOstar^®^ plate reader (Omega). The software determined fluorescence with 485 nm excitation and 520 nm emission wavelengths as well as the OD_600_. Relative fluorescence units (RFU) were normalized to the respective OD_600_.

### 4.6 Preparation of whole cell lysates (WCLs)

To obtain WCLs of *E*. *coli* and *V*. *cholerae*, cultures were grown in LB ON or inoculated in fresh LB to an OD_600_ of 0.1, grown to mid‐log phase followed by induction with 0.05% arabinose or as specified of plasmid‐derived expression for at least 2 h with aeration at 37°C, and 180 rpm shaking. Cell equivalents of 1.5 OD_600_ units were harvested by centrifugation in an Eppendorf centrifuge (5 min at 6,500 ×g), resuspended in 60 µl Laemmli buffer (Laemmli, 1970), boiled for 30 min at 100°C and used for standard sodium dodecyl sulfate‐polyacrylamide gel electrophoresis (SDS‐PAGE). WCLs were normalized to similar protein amounts by SDS‐PAGE Kang staining (Kang et al., 2002) before being used for immunoblot analysis.

### 4.7 SDS‐PAGE and immunoblot analysis

The standard SDS‐PAGE procedure was performed using 15% polyacrylamide gels (Mini‐PROTEAN^®^ Tetra cell, BIO‐RAD) in combination with the PageRuler^TM^ Prestained Protein Ladder (10–180 kDa, Thermo Fisher Scientific) as a molecular mass standard for separated proteins. Protein bands were stained according to Kang et al. (2002) or transferred to an Amersham^TM^ Protan^TM^ 0.45 µm nitrocellulose membrane (GE Healthcare Life Sciences) for further immunoblot analysis. After blocking in tris‐buffered saline (TBS) with 10% skim milk at 4°C ON, the membranes were incubated with the respective primary antibody at RT for 2 h: α‐ToxR rabbit serum diluted 1:1,000 in TBS 10% skim milk (Fan et al., 2014), kindly supplied by Jun Zhu, University of Pennsylvania, USA; α‐OmpU mouse serum diluted 1:5,000 in TBS 10% skim milk (Salem et al., 2015). After several washing steps as previously described by Fengler et al. (2012), the membranes were incubated with the respective secondary antibody at RT for 1 h: horseradish peroxidase conjugated goat anti‐rabbit (Dianova GmbH) diluted 1:10,000 in TBS 10% skim milk and horseradish peroxidase conjugated goat anti‐mouse (Dianova GmbH) diluted 1:7,500 in TBS 10% skim milk. The membranes were incubated in ECL solution for 5 min (Clarity^TM^ Western ECL Blotting Substrates, BIO‐RAD) for visualization of reactive protein bands by chemiluminescence using a Molecular Imager ChemiDoc^TM^ XRS system (BIO‐RAD). To adjust the protein content of different WCLs, final SDS‐PAGE was performed also using the same sample volumes for immunoblot analysis.

### 4.8 qRT‐PCR analyses

Oligonucleotides used for qRT‐PCR are listed in Table 2 and are labeled by x_fw or x_rv, whereas x represents the respective tested gene. qRT‐PCR was performed as described previously (Schild et al., 2007). RNA was isolated from a minimum of three independent cultures grown in LB medium until mid‐log phase (OD_600_ = 0.5) using QIAGEN^TM^ RNAprotect kit (Qiagen). To remove chromosomal DNA, the samples were treated with RQ1 RNase‐Free DNase (Promega). By using iScript^TM^ Select cDNA Synthesis Kit (Bio‐Rad), cDNA was synthesized from 200 ng RNA, also including controls without reverse transcriptase. Quantification of cDNA was performed with SYBR GreenER^TM^ qPCR SuperMix for ABI PRISM® instrument (Invitrogen) using a Rotor‐GeneTM 600 and Rotor‐GeneTM 600 Series Software 1.7 (GenXpress). Each reaction contained 400 nM oligonucleotides and 10 ng cDNA template. Each independent sample was tested in triplicates. Subsequently, the mean cycle threshold of the test transcript was normalized to the reference gene, *rpoB* and to one selected *rpoB* WT reference. Values above or below 1 indicate an up‐ or downregulation in the mutant, respectively, compared to the WT.

### 4.9 Statistical analysis

Normally distributed data were analyzed by the unpaired Student’s *t* test in case of single comparison. Ordinary one‐way ANOVA followed by either Dunnett’s or Sidak’s multiple comparison tests were conducted, respectively, for multiple comparisons.

In case of non‐Gaussian distributed data Kruskal–Wallis followed by Dunn’s multiple comparison tests were performed. Differences were considered significant for *p* values of < .05. GraphPad Prism version 7 was used for all statistical analysis.

## Conflict of Interest

The authors declare that the research was conducted without any commercial or financial relationships that could be constructed as a potential conflict of interest.

## AUTHOR CONTRIBUTIONS

*Designed the study*: Pennetzdorfer, Höfler, and Reid. *Performed the experiments and/or the analysis*: Pennetzdorfer, Höfler, Wölflingseder, and Tutz. *Contributed to the discussion and the data evaluation*: Pennetzdorfer, Höfler, and Reid. *Performed data analysis*: Schild, Pennetzdorfer, and *Wrote the manuscript*: Reid and Pennetzdorfer and Reid.

## Supporting information

Supplementary Material
