# Supplementary material for: σE controlled regulation of porin OmpU in Vibrio cholerae
Source: Mol Microbiol. 2021 Jan 25;115(6):1244–61. doi: 10.1111/mmi.14669 (PMC8359247; doi:10.1111/mmi.14669)
Supplement: Supplementary file 1 — Supplementary Material [file MMI-115-1244-s001.pdf]

Supplemental figures

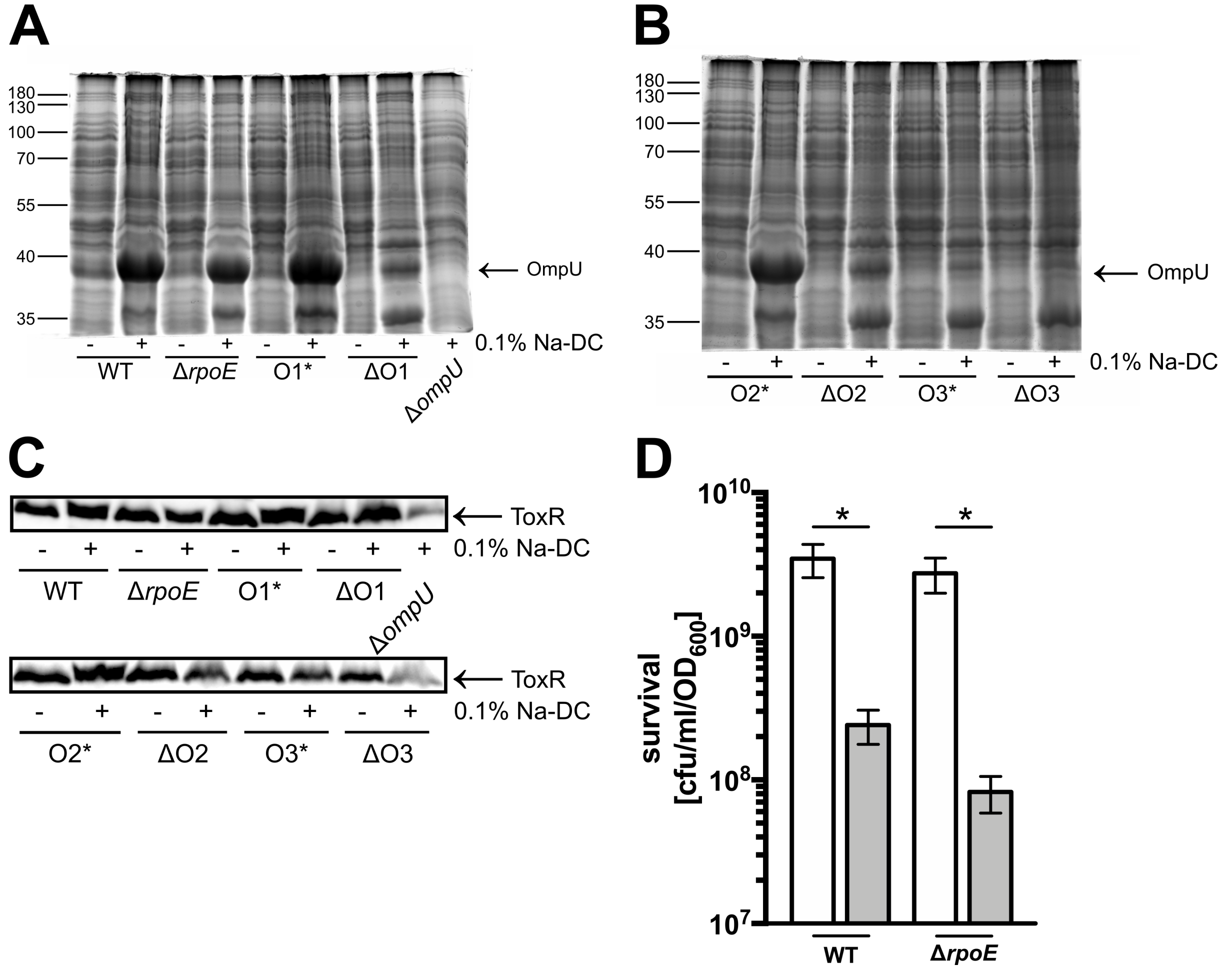

Figure S1: Comparative analysis of protein amounts and OmpU protein levels obtained from Na-DC treated cultures. **A**. Kang stained polyacrylamide gel of WCLs derived from WT, Δ*rpoE*, *ompU*O1*, *ompU*ΔO1 and Δ*ompU* grown in LB supplemented without or with 0.1% Na-DC ON. **B**. Kang stained polyacrylamide gel of WCLs obtained from *ompU*O2*, *ompU*ΔO2, *ompU*O3*, and *ompU*ΔO3 grown in LB supplemented without or with 0.1% Na-DC ON, serving as loading controls for immunoblots in Figure 2. **C**. Immunoblot analysis shows WCLs gained from WT, Δ*rpoE*, *ompU*O1*, *ompU*ΔO1, *ompU*O2*, *ompU*ΔO2, *ompU*O3*, *ompU*ΔO3 and Δ*ompU* utilizing α-ToxR antibodies grown in LB supplemented without or with 0.1% Na-DC ON (For a representative loading and quality control, see Kang-stained polyacrylamide gels in Figures S2AB). **D.** Survival plating of WT and Δ*rpoE* was

performed after incubation in LB (open bars) or in LB supplemented with 0.1% Na-DC (grey bars) ON. Data represent mean values ± standard deviation of 3 biological and 6 technical respectively, resulting in a total sample size of 18. Significant differences are marked by an asterisk (one-way ANOVA followed by a Sidak's multiple comparisons test, *$P < .05$; for a representative).

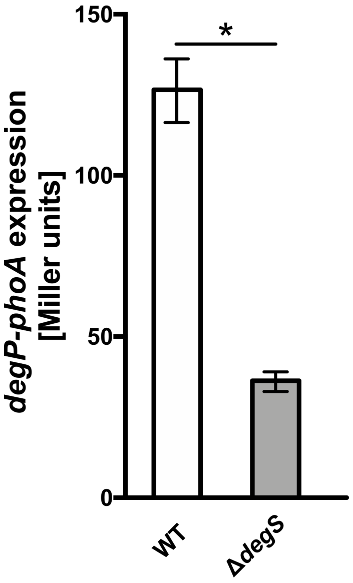

Figure S2: *degP* expression is impaired in Δ*degS*. Alkaline phosphatase activities (Miller units) were quantified in WT and Δ*degS* harbouring a *degP-phoA* transcriptional fusion grown in LB ON. Data represent mean values ± standard deviation of 6 biological replicates and a total sample size of 12. Significant differences between the WT and Δ*degS* are indicated by an asterisk (Student's *t* test, *$P < .05$).

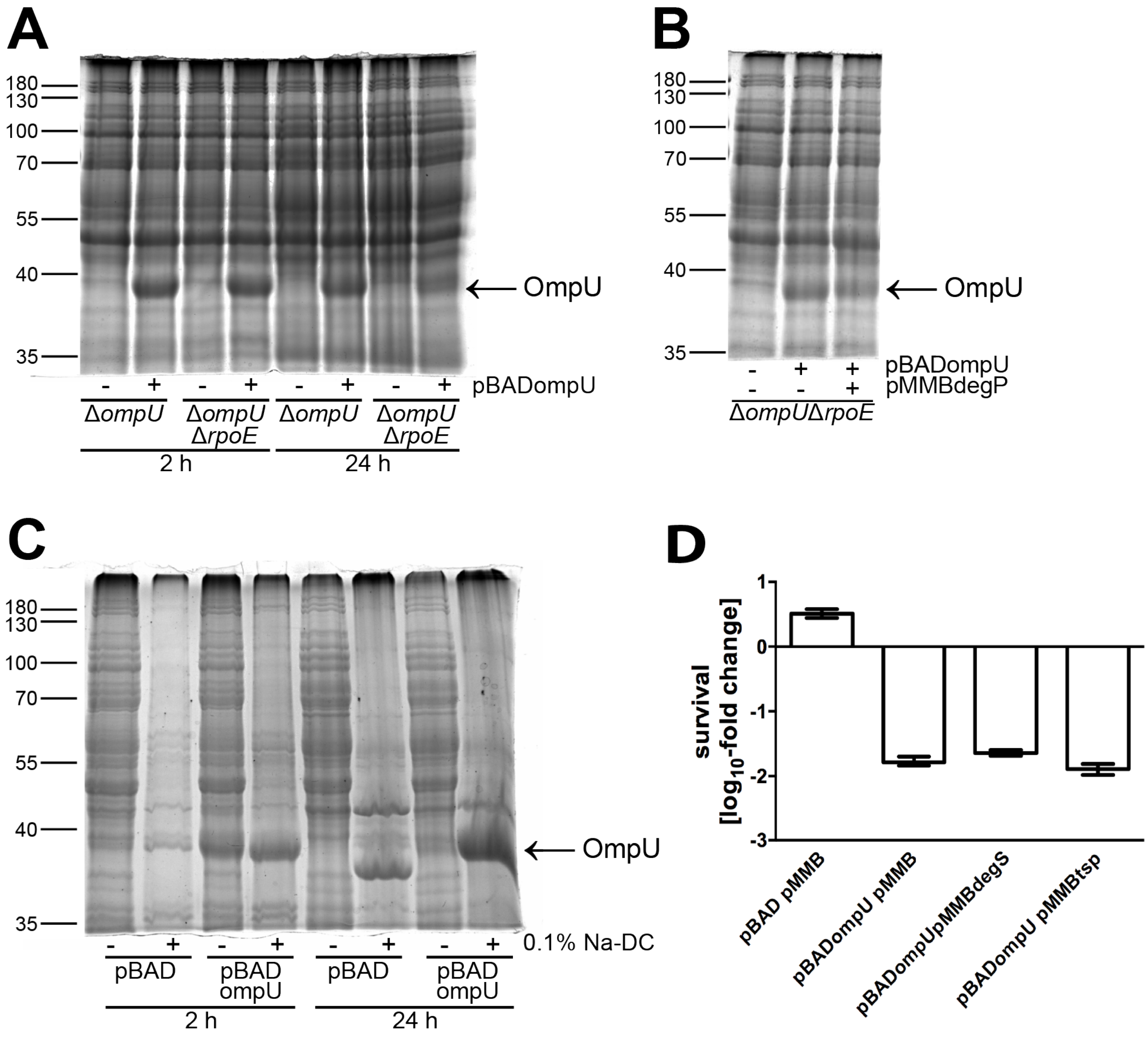

Figure S3: *ompU* expression control in Δ*ompU* and Δ*ompU*Δ*rpoE*. **A**. Kang stained polyacrylamide gel of WCLs gained from Δ*ompU* and Δ*ompU*Δ*rpoE* harbouring pBADompU or the empty vector control respectively in LB at time points 2 h and 24 h after induction of expression with 0.05% arabinose. **B**. Kang stained polyacrylamide gel of WCLs gained from Δ*ompU*Δ*rpoE* harbouring pBADompU, pMMBdegP or the empty vector control respectively in LB at time points 2 h after induction of expression with 0.05% arabinose and 1 mM IPTG. **C**. Kang stained polyacrylamide gel of WCLs gained from Δ*ompU*Δ*rpoE* harbouring pBADompU or the empty vector control in LB supplemented without or with 0.1% Na-DC at time points 2 h and 24 h after induction of expression with 0.05% arabinose. **D**. Log10 fold change presentation of survival plating of time point 2 h compared to time point 0 h obtained

from ΔompUΔrpoE harbouring pBAD and pMMB, pBADompU and pMMB, pBADompU and pMMBdegS or pBADompU and pMMBtsp respectively was conducted after simultaneous induction of plasmid-derived expression with 0.05% arabinose and 1 mM IPTG in mid-log phase. Data represent median with interquartile range of 4 biological or technical replicates.

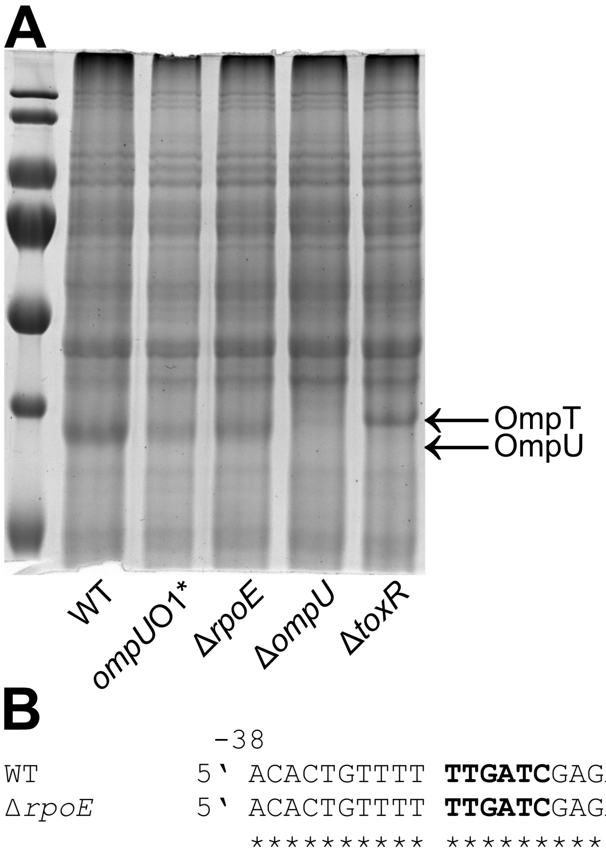

B

```
              -38                                            +1
WT        5' ACACTGTTTT TTGATCGAGA TTGGATTATT CTAAGTCTGC 3'
ΔrpoE     5' ACACTGTTTT TTGATCGAGA TTGGATTATT CTAAGTCTGC 3'
             ********** ********** ********** **********
```

Figure S4: Loading control for ToxR- and OmpU-immunoblots and *toxR* promoter region. **A**. Kang stained polyacrylamide gel of WCLs obtained from WT, *ompU*O1*, Δ*rpoE*, Δ*ompU* and Δ*toxR* grown in LB ON for immunoblots in Figure 4B. **B**. DNA sequence (5' – 3') of *toxR* promoter from position -38 to +1 relative to the transcription start site +1 of WT and Δ*rpoE*. Compliant nucleotides are indicated with asterisks. -35 and -10 regions are bold.

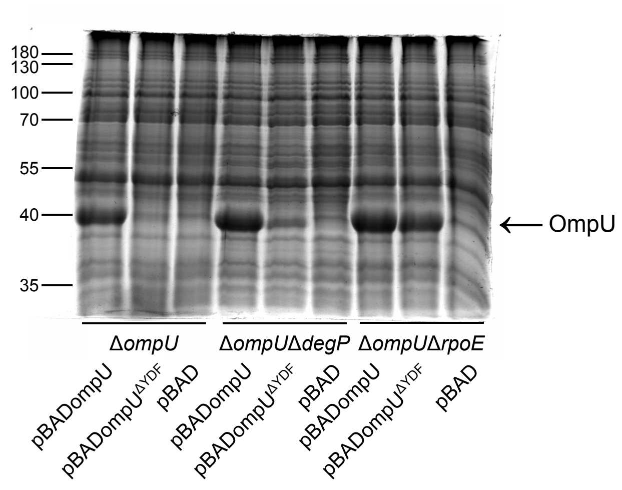

Figure S5: Loading control for OmpU-immunoblot. Kang stained polyacrylamide gel of WCLs were derived from Δ*ompU*, Δ*ompU*Δ*degP* and Δ*ompU*Δ*rpoE* carrying pBAD, pBADompU or pBADompU$^{\Delta YDF}$ respectively. WCLs were prepared after 2 h of plasmid-derived expression induction in LB.

```
Consensus               QFXXLXVKAS YRFADRXX-- XX----XXXX XXXYXDNGXD GYSLSAIYXX XXTGXXXGXG YADQDXXNEY
V. cholerae             QFGDLGVKAS YRFADRNAVD AMGNVVTETN AAKYSDNGED GYSLSAIYTF GDTGFNVGAG YADQDDQNEY 243
V. parahaemolyticus     QFENLSVKAS YRFADRK--- ------LNDA GTEYTDNGQD GYSLSAIYAV ADTGLELGAG YADQDEANEY 219
V. vulnificus           QFDALSVKAS YRFADRAD-- SS----KNNV DNTYIDNGKD GYSLSAIYAI GQTGLTLGGG YADQDKSNEY 214

Consensus               MLAASYXMXX LYFAGXFTDG EXX-XXXX-- ---------D YTGYELAXXY XXGQXXFTXT YNNAETXXXT
V. cholerae             MLAASYRMEN LYFAGLFTDG ELA-K-DV-- ---------D YTGYELAAGY KLGQAAFTAT YNNAETAKET 300
V. parahaemolyticus     MLAASYTMGD LYFAGIFTDG EKA-KTEG-- ---------D YTGYELAGAY TLGQTVFTTT YNNAETNNET 277
V. vulnificus           MLAASYTMGD LYFAGVFTDG EKDYGTNGDY SHRGFSSVED YTGYELAAKY TMGQTVFTTT YNNAETDGYT 286

Consensus               SXXNFAXDAX YYFKPNFRXY XSYXFNLXDX XXKXG----X XXXXKXXXED ELAXGLRYDF
V. cholerae             SADNFAIDAT YYFKPNFRSY ISYQFWLLDS D-KVG----- ----KVASED ELAIGLRYDF 350
V. parahaemolyticus     SANNFAVDAS YYFKPNFRGY VSYNFWLIDS GDKLGKVGGN TTASKADAED ELALGLRYDF 337
V. vulnificus           STDNFAVDAT YYFKPNFRGY VSYNFWLLDA GDKIG----T STISKADAED ELALGLRYDF 340
```

Figure S6: YXF motif conservation among *Vibrio* species. Multiple amino acid sequence alignment in ClustalW format of the C-terminal portions of OmpU which were analysed *in silico* in *V. cholerae*, *V. parahaemolyticus*, *V. vulnificus* resulting in a mutual consensus sequence. The YXF motifs are highlighted in bold. Respective gaps within the amino acid sequences are displayed by hyphens. The alignment was created with the M-coffee web server (Moretti et al., 2007).
